# Mapping and validating stem rust resistance genes directly in self-incompatible genetic resources of winter rye

**DOI:** 10.1007/s00122-021-03800-7

**Published:** 2021-03-10

**Authors:** Paul Gruner, Anne-Kristin Schmitt, Kerstin Flath, Hans-Peter Piepho, Thomas Miedaner

**Affiliations:** 1 State Plant Breeding Institute, University of Hohenheim, 70593 Stuttgart, Germany; 2 Institute for Plant Protection in Field Crops and Grassland, Julius-Kuehn Institute, Stahnsdorfer Damm 81, 14532 Kleinmachnow, Germany; 3 Biostatistics Unit, Institute of Crop Science, University of Hohenheim, 70593 Stuttgart, Germany

## Abstract

**Key message:**

Individual stem rust resistance genes could be directly mapped within self-incompatible rye populations.

**Abstract:**

Genetic resources of rye (*Secale cereale* L.) are cross-pollinating populations that can be highly diverse and are naturally segregating. In this study, we show that this segregation could be used for mapping stem rust resistance. Populations of pre-selected donors from the Russian Federation, the USA and Austria were tested on a single-plant basis for stem rust resistance by a leaf-segment test with three rust isolates. Seventy-four plants per population were genotyped with a 10 K-SNP chip. Using cumulative logit models, significant associations between the ordinal infection score and the marker alleles could be found. Three different loci (*Pgs1*, *Pgs2*, *Pgs3*) in three populations were highly significant, and resistance-linked markers could be validated with field experiments of an independent seed sample from the original population and were used to fix two populations for resistance. We showed that it is possible to map monogenically inherited seedling resistance genes directly in genetic resources, thus providing a competitive alternative to linkage mapping approaches that require a tedious and time-consuming inbreeding over several generations.

**Supplementary Information:**

The online version contains supplementary material available at 10.1007/s00122-021-03800-7.

## Introduction

Rye (*Secale cereale* L.) is a cross-pollinating crop typically appearing as self-incompatible populations. In theory, gene and genotype frequencies remain constant over generations in large random mating populations without selection (Hardy–Weinberg equilibrium, HWE). In breeding populations, however, crossing and selection, as well as random drift due to limitations on population size, will change gene frequencies. Gene frequencies will almost never be increased to one by phenotypic selection, leaving aside an idealized scenario of selection with 100% intensity on a single recessive gene with perfect penetrance (p.28f, Falconer and Mackay 2008). Consequently, populations are a reservoir of rare alleles, and a higher diversity (variation) within populations than across populations can be expected and has been observed in rye, for example for resistance to ergot severity (Mirdita et al. 2008). Here, we want to exploit this within-population variation for mapping stem rust resistance directly in self-incompatible populations of winter rye.

Stem rust (*Puccinia graminis* f. sp. *secalis*) can cause yield losses up to 60 percent in continental areas (Solodukhina and Kobylyansky 2001) and is thus considered an important disease in rye. In Central Europe, a high infection level cannot be observed in every season, but under suitable conditions (warm and dry summers) the fungus can spread over large areas. Severe stem rust outbreaks in rye were reported from North-eastern Europe (the largest growing region worldwide), Brazil (Roelfs 1985) and South Africa (Boshoff et al. 2019).

We chose five rye donor populations that were found to have stem rust resistance in previous field experiments on a single-plant basis (Miedaner et al. 2016). An additional criterion for our choice were the different geographical origins to increase the chance to find different resistance genes and further information that was available. Two populations from Russia, ‘HY2407/87′ (HY2407) and ‘HY75/81′ (HY75), consisted of pre-breeding material which was previously selected for leaf and stem rust resistances. Two Austrian landraces, ‘Oberkärntner’ being a selection from the ‘Lurnfelder Roggen’ and ‘Tiroler’, were historically associated with stem rust resistance and originated in the Austrian stem rust hot spots Lurnfeld and Tirol (Hänsel 1958; Schilperoord 2012). Another population, ‘Wrens Abruzzi’, was a descendant from Italian rye (‘Abruzzi’), which was introduced as fodder rye in the USA in 1900 and 1913 (Morey 1970). For the cultivar itself no information about stem rust resistance could be found, however in another strain of the Abruzzi rye, Mains (1926) detected stem rust resistant plants.

To phenotype the resistance, we used a seedling test, namely a detached leaf-segment test (LST) where detached leaves were inoculated and infection types were later assessed. The LST has already been used in other studies with rye (Miedaner et al. 2016; Gruner et al. 2020) and has the advantage that different rust isolates can be tested separately on the same plant. Similar infection types can be achieved on the detached leaves compared to whole-plant seedling tests (K. Flath, unpublished data) that are commonly applied to characterize (stem) rust resistance genes in wheat (McIntosh et al. 1995). Further, as the seedling plants remain unaffected and healthy, DNA can be extracted after performing the test from newly emerging leaves. Representative rust isolates were chosen from previous collections (Miedaner et al. 2016). They were characterized by a set of rye inbred lines (differential lines) showing different resistance patterns for different isolates.

As the LST was based on rye seedlings, we were limited to identify seedling resistances. Generally, we can distinguish between seedling resistance (= all-stage resistance), expressed by single resistance (R)-genes with large effects in all plant stages, and adult-plant resistance. Tan et al. (1976) also reported genes that were active in the seedling stage only. In a previous mapping study (Gruner et al. 2020), both all-stage and adult-plant resistances were identified in rye. Specifically, a R-gene candidate, *Pgs1*, firstly identified in the field was resistant against several isolates in the LST, too. More resistance genes from rye landraces were reported (Tan et al. 1976, 1977; Solodukhina and Kobylyansky 2000), showing the high potential of genetic resources. For example, Tan et al. (1976, 1977) found six and eight resistance genes against *Puccinia graminis* f.sp. *graminis* and f.sp. *secalis*, respectively, within inbred populations developed from four genetic resources from the USA and Kenya: Kenya, Wrens, Elbon and Gator. To the best of our knowledge, none of the previously identified resistances, except *Pgs1*, could be clearly linked with a chromosomal locus or molecular marker.

Based on genotyping by a SNP chip, we considered the marker-wise significance testing for association between phenotype and genotype as most appropriate. The statistical model of choice was a cumulative logit model for multinomial counts (Agresti 2019), accounting for the ordered infection type (IT) categories defined by the LST, and providing an asymptotic significance test for marker-trait associations. However, we also compared results with a non-parametric test (Konietschke et al. 2015) because sample sizes and counts for certain infection types in some populations were very small. So far in rye, only QTL mapping studies based on (biparental) populations composed of inbred lines were applied for agronomic traits (Miedaner et al. 2012; Hackauf et al. 2017) and fungal diseases (Wehling et al. 2003; Roux et al. 2004; Gruner et al. 2020). Inbreeding is possible due to a self-fertility gene, and today more hybrid than population cultivars can be found on the German seed market (Bundessortenamt 2019). Still, genetic resources (populations) are necessary for breeding of both cultivar types. The identification of resistance-linked molecular markers directly from genetic resources could speed up and simplify the introgression of new genes into the breeding progress.

We investigated the suitability of self-incompatible rye populations for mapping stem rust resistance genes and validated the most closely linked markers by a KASP assay and a field test. Our results are presented in the order of the workflow (Figure S1): The populations were (1) phenotypically analyzed by LST, (2) genotyped and (3) genotypic and phenotypic data was combined to find significant associations. To validate the results of (3), new seeds from the same populations were (4) genotyped with marker candidates, (4A) tested on single-plant basis in inoculated field trials and (4B) resistant plants were selected based on markers, intercrossed in isolation cabins and the offspring thereof again tested in inoculated field trials in the following year.

The milestones of this work were a proper statistical analysis of ordered ITs and mapping of gene loci in cross-pollinating winter rye populations and an independent validation of resistance in field trials.

## Material and methods

### Rye populations

Five rye genetic resources were investigated: The landraces Oberkärntner (OK) and Tiroler (TI) from Austria, Wrens Abruzzi (WA) from the USA, and two populations from Russia, HY2407/87 (HY2407) and HY75/81 (HY75), that were improved for rust resistances (stem and leaf rust) by mass selection and were received from the Research and Development Institute of Agriculture of Central Regions of the Non-Chernozem Zone of the Russian Federation (A. A. Goncharenko) in Nemchinovka near Moscow. Seeds from all other populations were originally obtained from gene banks. Populations were maintained at the University of Hohenheim over the last decades under cold storage and by propagating them every 15 to 20 years in isolation cabins (Geiger and Miedaner 2009). Isolation cabins covered a square of about 1.25 m length each side and were planted with about 60 to 90 plants in each propagation step. The last propagation was done in 2009 (HY2407, HY75, WA) and 2015 (OK, TI).

### Leaf-segment test

From each population, 100 rye seedlings were grown under rust-proof conditions for 10 days at 17 °C and continuous (24 h/d) light in a climate chamber. Ten days after sowing, the primary leaf of each plant was cut into three 3 cm long pieces and placed in different multidishes (squared petri dish separated into 15 segments) with water agar (6 g l^−1^) containing 35 mg l^−1^ benzimidazole and 1.5 mg l^−1^ silver nitrate. Each of the three leaf pieces from a single plant was inoculated with a different isolate (separate multidishes). In each multidish, three additional leaves from the susceptible cultivar ‘Palazzo’ (KWS LOCHOW GMBH) were placed serving as check. The isolates used for inoculation were collected in Germany in the past and could be traced back to a single pustule (Miedaner et al. 2016). All isolates showed a different pattern of resistance/virulence reactions on 15 differential lines also developed in a previous study (Table S1). The inoculation was carried out by means of an infection tower (Figure S2). Specifically, urediniospores previously multiplied on rye seedlings (cv. ‘Palazzo’) were mixed (each isolate separately) with talcum powder in a ratio of 1:3 and evenly blown onto multidish plates with leaf segments. For comparability, the same isolate-talcum mixture was used for all materials analyzed for this study. Thereafter, leaf segments were stored for 24 h at 100% humidity, 20 °C and darkness followed by continuous light at 20 °C. After 14 days, the infection type was visually assessed on an ordinal rating scale described next.

### Rating scheme

Using the rating scale of Stakman et al. (1962) as a template, the infection type (IT) was assigned to the following categories:

0 = No uredinia or indications of infection.

1 = No uredinia but hypersensitive reaction.

2 = Small uredinia with necrosis.

2.5 = Small to medium-sized uredinia on green islands surrounded by necrosis and chlorosis.

3 = Mid-size uredinia with or without necrosis.

4 = Large uredinia without necrosis.

In contrast to the original scale and scale interpretations (Stakman et al. 1962), we considered IT ≤ 2 as resistant and IT > 2 as susceptible reaction. This change of the score ‘2.5′ into the susceptible category was led by the susceptible check variety that was scored as ‘2.5 ‘or ‘3' in the experiments. For the mapping procedure described later, the categorization into resistant and susceptible was irrelevant due to statistical reasons.

### Choice of plants for DNA analysis

From each population, 100 plants were assessed by LST. We chose 74 plants from each population for DNA extraction. We removed plants where the IT could not be assessed or the leaves of the check remained uninfected, and tried to shift the ratio of resistant (IT ≤ 2 and susceptible plants (IT > 2) toward one aiming for equal group sizes and increase in detectability and statistical power of rare alleles.

### Marker analysis

The DNA extraction from leaf samples and genotyping was done with a rye 10 K Infinium iSelect SNP chip proprietary to and at KWS SAAT SE & Co. KGaA. The SNPs of this assay were partially overlapping with the 5 k-SNP assay of Martis et al. (2013) and the 600 k-SNP assay of Bauer et al. (2017) so that 2515 SNP markers could be located on a linkage map constructed by the latter. For each population, markers were filtered to have a minor allele frequency (MAF) > 5% and less than 5% missing values. The genotyping procedure was not successful for three plants from HY75 and one from HY2407.

### Principal coordinate analysis (PCA)

To perform a principal coordinate analysis, only markers were used that had less than 5% missing values after all populations were combined. Marker data were coded as 0, 0.5, 1 (homozygous for one allele, heterozygous, homozygous for the other allele) and stored in a matrix *C* = {*c*_*ij*_} with *n* individuals and *q* markers. Before calculation of the genetic covariance matrix $X=\frac{1}{n}M{M}^{^{\prime}}$ and decomposition thereof into eigenvectors, the entries of *C* were corrected by the column mean ${\stackrel{-}{c}}_{\bullet j}$ for the *j*-th marker and the estimated allele frequency ${p}_{j}={\stackrel{-}{c}}_{\bullet j}/2$ resulting in the standardized marker matrix *M* = {*m*_*ij*_} with ${m}_{ij}=\frac{{c}_{ij}- {\stackrel{-}{c}}_{\bullet j}}{\sqrt{{p}_{j}(1-{p}_{j})}}$ (Patterson et al. 2006). The calculation of the covariance matrix and decomposition into eigenvectors was done with the R functions cov() and eigen() (R Core Team 2019). The variance proportion explained by the first two components was calculated by dividing the eigenvalue of the respective component by the sum of all eigenvalues. As shown by PCA, three genotypes (= three plants) from population HY2407 were located in the cloud of HY75 and vice versa and thus were discarded from further analysis.

### Linkage map

No physical reference map for the used markers was available, and a public linkage map (Bauer et al. 2017) did only overlap with 2515 markers. Thus, a consensus map was constructed using four linkage maps from previous projects (Gruner et al. 2020), five linkage maps from new biparental populations (*n* = 91, data unpublished), and the overlap of the linkage map published by Bauer et al. (2017). The consensus map was built with the MergeMap online tool (Wu et al. 2008). From the filtered markers used in this work 6863 markers could be placed on the consensus map. The remaining non-overlapping markers were still considered for the mapping procedure described in the following section and for results display summed up in the artificial linkage group “unmapped” with arbitrary positions.

### Statistical analysis

#### Phenotypic model

To test whether within a single population the isolates show significantly different reactions and to assess the genetic variance in the populations, a cumulative logit mixed model (clmm) was fitted. Following the notation of Agresti (2019, pp. 284), it can be written as:

1 $$ logit\left[ {P\left( {Y_{ik} \le j} \right)} \right] = g_{i} + \alpha_{j} + \beta_{k} $$

In words, the log odds (logit) of the response ${Y}_{ik}$ to fall in category $j$ (*j* = 1, …, 6) or below were modeled by the random effect ${g}_{i}$ for the $i$ th genotype (= grouping factor or cluster = plant) and the fixed effect ${\beta }_{k}$ of the isolate $k$. Whereas for each isolate *k* a separate effect was estimated, the fixed intercept ${\alpha }_{j}$, also known as threshold, depended on the different ordinal infection categories $j$ of the response variable. The response variable was transformed into an ordered factor with factor levels from one to six.

The clmm was fitted with the clmm() function of the R package ordinal (Christensen 2019; R Core Team 2019) using full maximum likelihood estimation by setting the nAGQ option (the number of quadrature points to use in the adaptive Gauss-Hermite quadrature approximation to the likelihood function) to 20. By defining the isolate as factor (in R language), no effect for the first level of the variable isolate was estimated and the estimated effects of the remaining isolates (factor levels) correspond to pairwise differences compared to the first factor level. All pairwise differences were thus estimated by successively recoding isolates so that each isolate in turn was the first factor level and the analysis provided all differences compared to that isolate. The variance estimated for the random effect ${g}_{i}$ was considered as genetic variance.

#### Mapping procedure

To test for significance, the phenotypic cumulative logit model (1) was extended by fixed codominant and dominant marker effects ${\gamma }_{a}$ and ${\gamma }_{d}$. For regressing the response on each marker *m*, the marker alleles for the codominant effect ${\gamma }_{a}$ were coded as ${m}_{a}=$ 0, 1, 2 (homozygous for the one allele, heterozygous, homozygous for the other allele) and for the additional dominant effect ${\gamma }_{d}$ the coding was ${m}_{d}=$ 0, 1, 0. The fitted model was:

2 $$ logit\left[ {P\left( {Y_{ikm} \le j} \right)} \right] = g_{i} + \alpha_{j} + \beta_{k} + \gamma_{a} m_{a} + \gamma_{d} m_{d} $$

The p-values for the marker effects ${\gamma }_{a}$ and ${\gamma }_{d}$ were extracted from the coefficient table in function call. Additionally, p-values for both marker effects simultaneously could be derived by comparison of model (2) with model (1) by anova()-function (R Core Team 2019). For calculation of test statistics, this function is based on the assumption that log-likelihoods of two nested models are asymptotically Chi-squared-distributed. Please note that the mapping procedure across isolates did not require genotypic means, instead we used the isolate-specific records as response and modeled the genotype (= plant) as random adjusting for the correlation of the isolate-specific records due to sampling from the same plant.

For markers being significant in clmm, the effect sizes ${\gamma }_{a}$ and ${\gamma }_{d}$, the odds of the sum of effects $\gamma$ (odds = $\mathrm{exp}(\gamma )$) and the ordinal superiority measure (OSM, Agresti and Kateri 2017) were reported. The OSM was calculated as $OSM \approx \frac{\mathrm{exp}(\gamma /\sqrt{2)}}{1+ \mathrm{exp}(\gamma /\sqrt{2)}}$ and gives the probability [0,1] that the values of the ordinal infection type (IT) are smaller for plants having marker allele zero compared to plants having marker allele one (or two). As this measure only gives a probability to reach smaller IT in general we additionally calculated OSM1 that defined the probability of being below a certain (predefined) IT. The most reasonable definition for resistant plant reactions was the absence of uredinia with chlorosis and necrosis (IT $\le$ 2) as well as the IT of the susceptible check (2.5 and 3) and thus the OSM measure was extended by the intercept ${\alpha }_{3}$ (IT ≤ 2), so that $OSM1 \approx \frac{\mathrm{exp}(({\alpha }_{3}+\gamma )/\sqrt{2)}}{1+ \mathrm{exp}(({\alpha }_{3}+\gamma )/\sqrt{2)}}$. To measure the proportion of genetic variance p_G_ that was explained by a marker fit, the difference between estimated genetic variance of model (2) and model (1) was divided by the genetic variance estimated from model (1). As an arbitrarily one of the two homozygous marker states was coded as 0 and the other as 2, the coding was switched when calculated effects of ${\gamma }_{a}$ and ${\gamma }_{d}$ had opposite signs. The sign of the effects did not affect the significance testing of the markers.

We aimed to fit additional effects for marker–isolate interactions, but for most of the markers the small sample sizes and low counts for certain IT categories impeded model convergence and thus the implementation of a standard routine over all markers. To not neglect the isolate-specific resistances, we fitted an isolate-specific model by sub-setting the data frame and applying model (2) without isolate effect ${\beta }_{k}$. Here, also the random genotype effect was dropped (one genotype = one isolate) and thus the cumulative logit model (clm) was fitted with the vglm () function from the VGAM package (Yee 2010). In the function call, the family option was set to “cumulative(parallel = TRUE)”. The p-values for the effects as well as the comparison of the full model with an intercept model could be calculated by applying the anova() function. Please note that in the following the abbreviation for models with random effect is clmm (cumulative logit mixed model) and for models without it is clm (cumulative logit model).

We additionally compared the isolate-specific testing based on clm with results from a non-parametric rank-based test similar to a “Dunnett” test but with user defined contrast matrices modeling the codominant and dominant marker effects from model (2). When markers were coded as factor levels 0, 1 and 2, the contrast matrix was defined as:

$$K= \left(\begin{array}{ccc}-1& 0& 1\\ 1& -2& 1\end{array}\right)$$

with the codominant contrast in the first and dominant contrast in the second row. The test was applied with the R package nparcomp and the mtcp() function (Konietschke et al. 2015).

#### Significance threshold

To adjust the significance threshold for multiple testing, we used the simpleM method proposed by Gao et al. (2008). The method was based on the principle of the Bonferroni correction, where the defined genome-wide significance threshold α is divided by the number of tests (markers) *q* in order to obtain the SNP-wise significance level. But instead of considering all markers, the number of *q* is reduced by PCA to *q*_*eff*_, the effective number of markers. As proposed by Gao et al. (2008), it was defined by the number of eigenvalues that explain 99.5% of the variation for SNP data. To run the PCA, the marker data were coded as 0, 1, 2, split into linkage groups and the correlation matrix was produced using the cor() function in R (R Core Team 2019) with the option use = "pairwise.complete.obs". The chromosome-wise calculated *q*_*eff*_’s were summed up to *q*_*eff_G*_ which was then used as divisor for α.

### KASP analysis and field validation

In autumn 2018, an independent seed sample (Table 1) was taken from every population and grown in multi-pot trays until the plants had enough tillers to be divided in two or three parts (vegetative clones). Leaf samples were taken for DNA extraction and analyzed with KASP markers that were developed from the respective markers of the SNP chip. From the plants divided in two parts, one clone served for validation in an artificially inoculated field trial (experiment A) and the other for crossing in an isolation cabin (experiment B). About 20 percent of the plants from each population (Table 1) were divided in and used as replicates in experiment A to assess the accuracy (correlation of scores) of single-plant ratings. The stem rust infection was visually assessed as percent affected stem surface between the leaf below the flag leaf and the node above that was covered with uredinio- or teleutospores. The trial was inoculated with an isolate mixture including the three isolates used in the LST plus two additional isolates (Table S1, for detailed experimental methods see Gruner et al. 2020). For experiment B, the remaining second (or third) clone was planted in an isolation cabin. This was a squared field plot that was covered by a polyethylene-foil cabin during flowering. It allowed vernalization and a vital plant growth in the field (compared to artificial greenhouse conditions) and isolated the clone from foreign pollen. After KASP analysis (before flowering) all clones not having the marker allele for resistance were removed, so that only single plants with homozygous resistance (marker) alleles mated with each other in the following summer. The number of plants investigated and the respective markers used can be found in Table 1. Flanking sequences for SNPs of KASP marker development can be found in Table S2. The marker isotig12934 was identified in a previous study (Gruner et al. 2020). From WA, no KASP marker was developed, but it was included in field experiments as the high frequency of susceptible plants (in LST) was considered useful as susceptible check. The seeds produced in isolation cabins were harvested in summer 2019, and about 120 kernels were sown in two rows in autumn 2019 in the field and again assessed in artificial inoculated field trials in 2020 using the same isolates as before.

**Table 1:** Sample sizes for leaf-segment test, genotyping, field validation and isolation cabins

Pop	*N* LST and geno	*N* field (exp. *A*) geno	*N* field (exp. *A*) rep	Marker	N isolation cabin
HY2407	71 (68)	108	21	isotig12934 + isotig12866	13
HY75	73 (70)	120	24	isotig12934	45
OK	74	120	24	C9750_251	–^a^
TI	74	66	16	isotig14536	12
WA	74	144	29	–	–

Number of plants (*N*) tested by the leaf-segment test and thereof (successfully) genotyped with the SNP chip (N LST and Geno, after outlier removal), used for validation in experiment *A* (exp. *A*) in the field (*N* Field Geno) and of plants that were replicated by dividing them into two pieces (*N* Field Rep) and the markers that were used to select *N* plants (vegetative clones) to mate in the isolation cabin (*N* Isolation cabin). For WA, no KASP was developed

^a^No result in KASP analysis

## Results

### Leaf-segment test

**Fig. 1:**
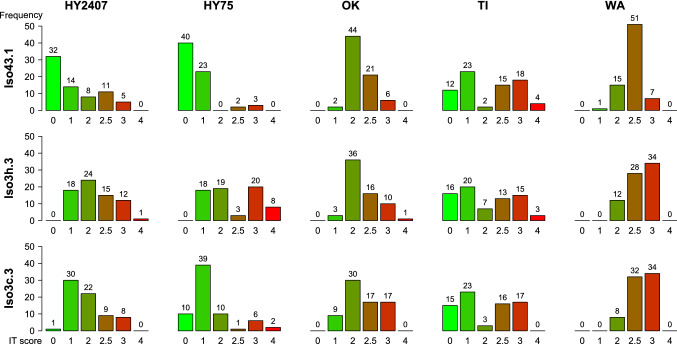
Frequency of different infection types for the three different isolates Iso3c.3, Iso3h.3 and Iso43.1 in the five populations HY2407, HY75, OK, TI, WA. Numbers on top of the bars are the exact counts for the respective categories

The observed virulence or resistance symptoms were assigned to all defined ITs. The susceptible check ‘Palazzo’ had ITs of 2.5 or 3. The distribution of the respective ITs differed between populations (Fig. 1). In WA, no IT of 0 or 4 was observed as well as no IT of 0 and only a single IT of 4 in OK. The populations HY75 and HY2407 showed a high amount of resistant plants (IT ≤ 2) and WA had mainly susceptible plants (IT > 2). For TI, two almost equally sized groups of resistant and susceptible plants could be found. The ratios displayed in Fig. 1 were based on the plants that were chosen for genotyping (*n* = 74). In the first step, 100 plants per population were analyzed by LST. The ratios of those plants were more unbalanced in terms of resistance (IT ≤ 2) and susceptibility (IT > 2, data not shown) and could not be equalized by choosing mainly plants having the minor IT (for WA: plants with IT ≤ 2, for HY2407, HY75, OK: plants with IT > 2). Within the populations, differences between the isolates could be observed (Fig. 1) and were significant for all populations except OK (Table 2). For TI and WA, only single comparisons between the isolates differed significantly. By the chosen model, we could also estimate the genetic variance for each population and it was highest in TI (Table 2). Differences between the populations could also be seen in the range of infection types.

**Table 2:** Comparison of virulence reactions of three isolates (Iso3c.3, Iso3h.3, Iso43.1) on the plants of five populations.

Population	Iso3h.3	Iso3c.3	Iso43.1	Var_G_	StErr(Var_G_)
HY2407	a	b	c	1.5	1.2
HY75	a	b	c	1.5	1.2
OK	a	a	a	3.1	1.7
TI	ab	a	b	15.8	4.0
WA	a	b	b	2.8	1.7

The significance was tested by a cumulative logit model with random genotype (= cluster) and taking the leaf-segment test-scores (0, 1, 2, 2.5, 3, 4) as ordered factor levels from one to six. Isolates were compared within populations (rows), and all isolates not sharing any letter are significantly different based on the Wald-test statistic at the 5% level of significance. Additionally the estimated genetic variance (Var_G_) with the standard error (StErr (Var_G_)) are reported

### Marker and population structure

**Fig. 2:**
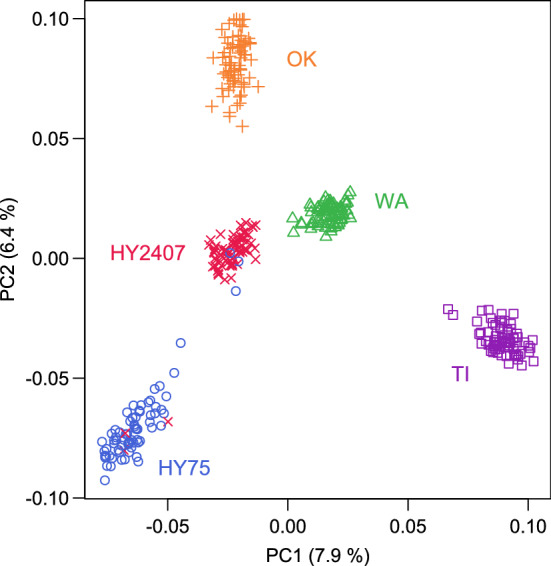
Principal coordinate analysis based on Eigen decomposition of a genetic covariance matrix calculated from marker-mean and -frequency adjusted SNP marker data. Genotypes from different populations are indicated by different color and symbol. The variance proportion (%) explained by the first component (PC1), and the second component (PC2) is given in brackets

A high amount of polymorphic markers (7641) remained from the 10 k chip after filtering (Table S3), and within the single populations 5708–6286 markers were polymorphic. However, the effective number of markers (*q*_*eff*_), used later to calculate the critical threshold in the mapping procedure, was estimated to be between 387 and 448 markers only. Heterozygosity ranged between 37 and 40 percent within the single populations, and the minor allele frequency was about 30 percent (Table S3). By using a correlation matrix and PCA, all populations could be clearly separated (Fig. 2) except three genotypes (= plants) from HY75 and HY2407 that were erroneously grouped. Because the same number of genotypes was outlying in both populations, they were considered as outliers and removed from further analysis. The overlap between HY75 and HY2407 could had been also been due to their similar origin.

### Mapping

**Fig. 3:**
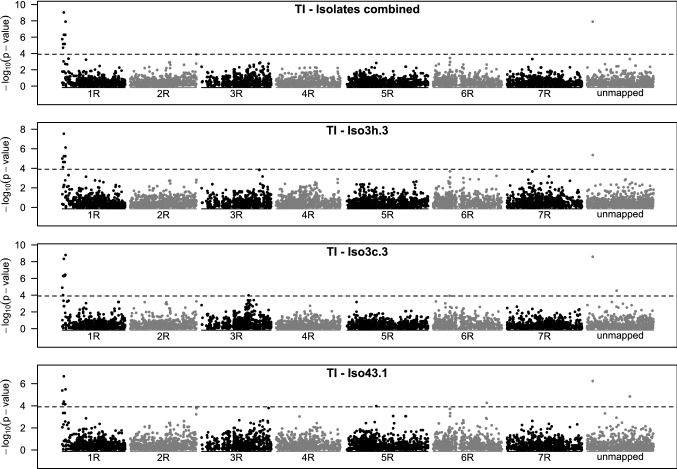
Manhattan plot for marker-wise significance testing of association between infection type and SNP marker score of population Tiroler (TI). A codominant (coded 0,1,2) and dominant (coded 0,1,0) marker effect was fitted simultaneously. p-values were based on ANOVA of the full model compared with a model without marker effects. The association was tested for all isolates combined (with fixed isolate effect and random genotype effect) and for all isolates (Iso3h.3, Iso3c.3, Iso43.1) separately

When testing markers for significant association, the p-value accounting for codominant and dominant marker effects simultaneously (comparison of model 2 with model 1) was most informative. We could find non-isolate-specific and isolate-specific associations between phenotype and marker (Fig. 3, Figure S3–Figure S6). Most markers being significant across isolates were also significant for some isolates, but not for all and not vice versa. In the following, population-specific results are reported. A summary can be found in Table 3 and contingency tables for the respective marker-IT combinations in Table S4–Table S19.

**Table 3:** Estimated model parameters:

Pop	Gene	Marker	Chr	Pos (cM)	Pos. Bauer (cM)	p-value codom	p-value dom	p-value codom + dom	Effect codom	Std. Effect codom	Effect dom	Std. Effect dom	Odds	OSM	OSM1	p_G_	n0	n1	n2
TI	*Pgs2*	isotig14536	1	25.7	7.2^b^	7.3E-07	1.9E-03	9.3E-10	– 2.9	0.6	– 2.5	0.8	228	0.98	1.00	0.48	29	32	13
HY2407	*Pgs1*	isotig10644	7	722.3	138.7^b^	1.7E-07	1.0E-01	1.2E-06	– 1.3	0.2	– 0.6	0.4	6	0.79	0.86	0.56	16	28	24
HY2407		isotig12866^a^	7	701.1	138.7	2.0E-05	2.5E-02	1.1E-04	– 1.3	0.3	– 0.9	0.4	9	0.82	0.92	0.39	9	31	30
OK	*Pgs3*	C9750_251	2	626.4	170.0	5.0E-02	8.2E-03	7.3E-06	– 0.9	0.4	– 1.4	0.5	10	0.84	0.97	0.42	29	40	5

For the single populations (Pop.) and by means of a cumulative logit model combining data from all isolates (fixed effect for isolate, random effect for genotype) the parameters estimated for the respective markers are reported: Chr. = chromosome; Pos. = position on consensus map; Pos. Bauer = position on the map of Bauer et al. (2017,) where (^b^) indicates that markers were not mapped by Bauer et al. (2017) and position of neighboring marker is reported instead; p-value, effect size and standard deviation of the effect size for a codominant (codom.) effect (marker coded 0,1,2) and dominant (dom.) effect (marker coded 0,1,0); p-value for the comparison (anova) of a model without marker effects and with both effects; the odds = 1/exp(effect codom. + effect dom.); OSM = ordinal superiority measure for the effect; OSM1 = ordinal superiority measure for the effect plus intercept of IT ≤ 2; pG = explained genetic variance; *n*0, *n*1, *n*2 = number of genotypes coded 0, 1, 2 (codominant) or 0,1,0 (dominant). (^a^) This marker was not the most significant at this locus but was applied as KASP marker

Associations in HY75 were not considered because almost all p-values were below the defined significance threshold (Figure S3) and the only four markers that could pass the threshold were almost completely heterozygous with only 3 to 6 homozygous alleles and could considered as genotyping errors that remained after marker filtering based on MAF and missing values. The results from WA (Figure S4) were not considered either, because the infection type was generally on a high level (Fig. 1) and not enough resistant plants could be included in the analysis. For TI the results were most conclusive. There, a single highly significant peak (several markers) could be observed on chromosome 1R (Fig. 3). The marker isotig14536 was the most significant for all isolates combined and for Iso3h.3 and Iso43.1 and also passed the threshold for Iso3c.3, where marker isotig19397 was most significant. If considered separately, both the codominant and dominant effects were highly significant and the similar effect sizes indicated that this gene was fully dominant (Table 3). Compared to other populations, this marker had generally the biggest effect size (Table 3) and other parameters that were estimated from the model further indicated a high association between marker alleles and LST. The presence of the resistance-linked marker allele (codominant + dominant) reduced the IT measured by LST in 98% of the cases compared to the non-resistance allele (OSM). If we consider only the reduction of the IT to “2” or smaller (expressed by OSM1) and thus account for the IT-specific intercepts of our model, 100% of plants carrying the resistance-linked marker allele were assigned to this category (Table 3). However, this single marker only explained 48% of the genetic variance. The remaining variance could have been explained by other (isolate-specific) genes or by (unknown) non-genetic effects. In addition to the peak on chromosome 1R, a single unmapped marker (Contig1811) was significant, but this again had the highest correlations with the significant markers from chromosome 1R, i.e., the correlation with isotig19397 was 0.66. Another peak with several significant markers could be found in population HY2407. It was located at the distal end of chromosome 7R (Figure S5). The marker isotig10644 was significant across isolates, and for Iso3c.3 and Iso43.1. No significant marker for Iso3h.3 could be found at this locus. The OSM of this marker was 79% and OSM1 of 86% (Table 3). Another significant marker (isotig12866) at this locus (across isolates and for Iso3c.3) yielded a higher dominance effect and could also reach a higher OSM of 82% and OSM1 of 92%. This marker was used as KASP marker for field validation. Here, the codominant and dominant marker effects were both highly significant and the dominant effect size was almost as high as estimated for the codominant (Table 3). Despite the smaller dominant effect of isotig10644, it explained more (56%) genetic variance than isotig12866 (39%). Further isolate-specific markers on chromosome 4R (Iso3h.3, Iso3c.3) and 6R (Iso3c.3) passed the significance threshold (Figure S5). In OK, only a single marker (C9750_251) was significant across isolates (Figure S6). It was also significant for Iso43.1 where additional significant markers could be found on chromosome 3R and 4R. Some located on chromosome 3R were also significant for Iso3c.3, however just below the threshold when isolates were combined. Special for this marker was that the codominant effect was smaller than the dominant effect that indicated overdominance; however, high standard errors of the effects did not prove this difference to be significant. Comparable with the markers reported before also here the OSM and OSM1 were high (84%, 97%), and the explained genetic variance was medium (42%, Table 3).

On the isolate-specific level, the p-values calculated by clm were compared with p-value derived from the non-parametric test (mtcp, Figure S7—Figure S11). Rather than having the exact same p-values, it was important that the same markers were significant for both methods. Nearly all markers that passed the threshold with the clm method also passed the threshold with the mtcp method and were even slightly smaller. Hence, we concluded that the assumption of a multinomial distribution that was made for clm and clmm test statistics was appropriate for our data and the sample sizes were sufficient to make similar conclusions compared to the exact non-parametric test. However, on average about eight times more markers at one to six additional loci per population and isolate were passing the threshold by mtcp method compared to clm so that it might be that some loci remained undetected with clm. However, listing and discussing all additional isolate-specific mapping signals was beyond the scope of this work.

### KASP analysis and field validation

The first prerequisite for validation of the markers was the successful conversion into KASP assays. Whereas the markers isotig12866 (HY2407) and isotig14536 (TI) were segregating in a newly taken plant sample, the marker C9750_251 (OK) did not produce any results when converted into a KASP assay. We further included marker isotig12934 for the analysis of HY2407 and HY75, because it was already converted into a KASP assay and shown to be linked to *Pgs1,* a gene at distal end of chromosome 7R and originally derived from HY75 (Gruner et al. 2020). In HY2407, two other markers were significant in the exact same chromosomal region according to the linkage map from Bauer et al. (2017).

**Fig. 4:**
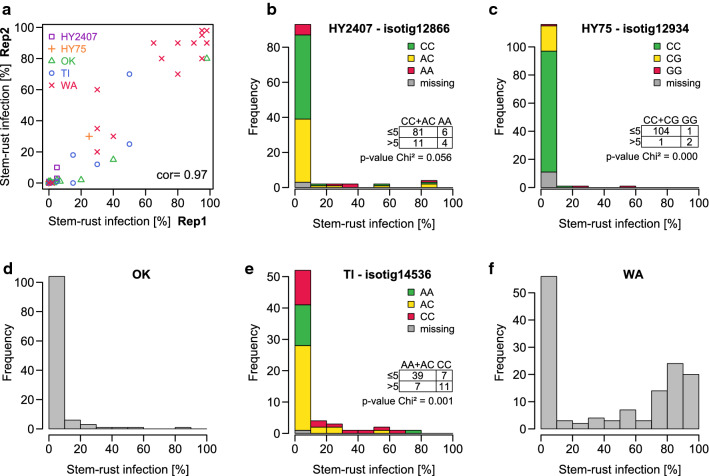
**a**. Stem rust infection of plants in the adult-plant stage from the populations HY2407, HY75, OK, TI and WA (colors and symbols) that were vegetatively cloned (divided in two pieces) and randomly placed on the field. Each piece was assessed (Rep1 and Rep2), and Pearson correlation (cor) was calculated. **b**–**f**. Histograms of the infection level of the different genotypes in the five populations HY2407, HY75, OK, TI and WA. For cloned plants with two replicates (one genotype = two clones), the mean was calculated. For the populations that were analyzed with a KASP marker, the respective marker is reported in the graph title and the bars are colored by the respective marker alleles. Thereby the green color stands for the resistance allele identified in the seedling stage. Genotypes were clustered in resistant (stem rust infection ≤ 5%) and susceptible (stem rust infection > 5%) and based on that clustering p-values were calculated by Pearson's Chi-squared test

In experiment A, the KASP-analyzed plants (Table 1) were scored for stem rust infection in artificially inoculated field trials. The infection of the single plants ranged between 0 and 98% stem rust severity (Fig. 4a), but the amount of resistant and susceptible plants differed highly between the populations (Fig. 4b-f). The comparison between the results from LST and the field scoring was difficult, not only because the field experiments were based on a new seed sample from the same population, but also because additional APR genes/QTLs could be active, seedling resistance could be inactive and we used two additional rust isolates for field inoculation. Thus, the comparison here focuses only on all-stage resistance genes that caused full resistance in the field (< 5% infection) as observed in other experiments with *Pgs1-*resistant material in the same location with the same rust isolates (data not shown). In HY75, only three percent of the plants (*n* = 4) showed a stem rust severity > 5%, whereas in WA 63% (*n* = 90) were infected > 5%. The populations HY2407, OK and TI also showed much more resistant than susceptible plants. When the marker allele was compared to the infection level, Chi-squared test statistic with grouping by an infection level ≤ 5% or > 5% for resistant and susceptible plants resulted in p-values < 0.05 for the markers in HY75 and TI and a p-value of 0.056 for the markers in HY75 (Fig. 4b, c, e). Plants classified as resistant by the marker allele and being susceptible in the field indicated an insufficient linkage between marker and resistance, a resistance gene being active in seedling stage only, or must be considered as an inaccuracy of the experiments. However, by replicating some self-incompatible genotypes (plants, Table 1) through vegetative cloning, assessments made on both clonal parts showed a very high correlation (*r* = 0.97, Fig. 4a). The observed resistance of plants that were classified as susceptible by the marker on the other hand could also been explained by additional resistance genes, especially by adult-plant resistance that could not be detected in the seedling stage. For example, WA showed a much higher number of resistant plants in the field than when testing at the seedling stage (Figs. 1, 4f).

**Fig. 5:**
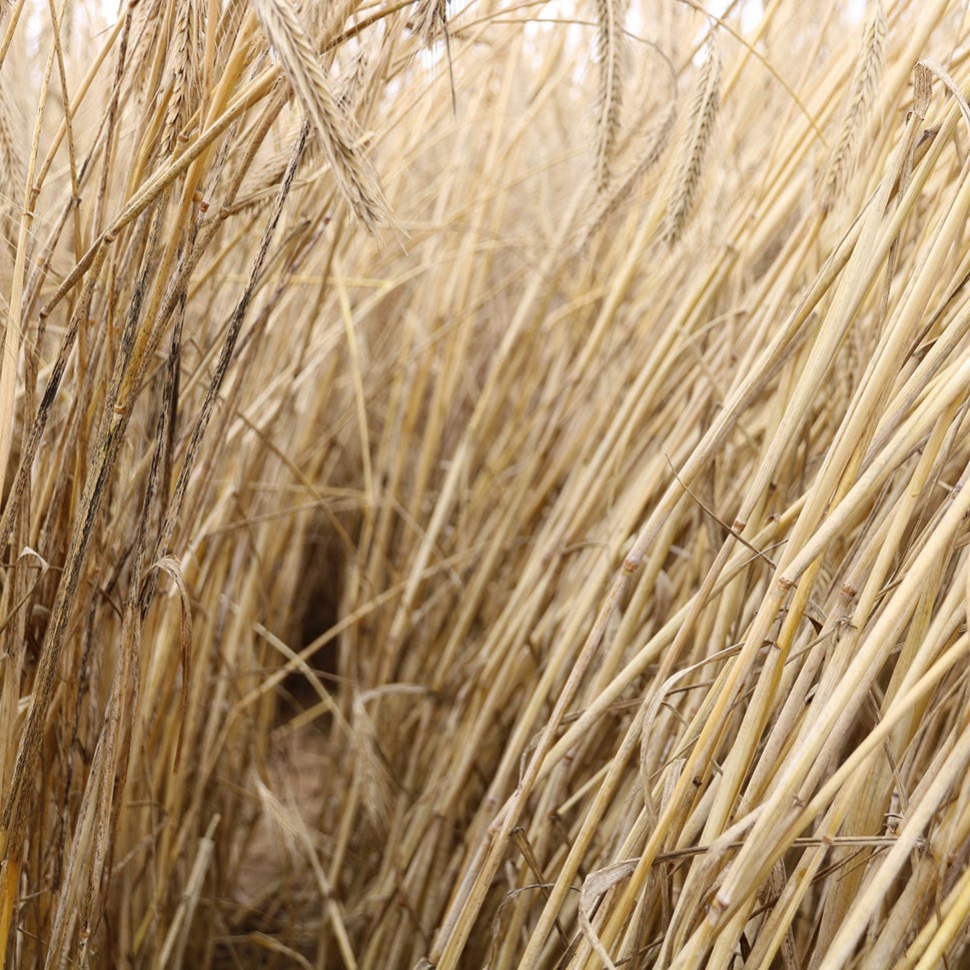
Offspring of the population TI where parents were selected for resistance with a KASP marker (isotig14536, right) compared to a susceptible single-cross hybrid (left). The picture was taken in an artificial inoculated field trial where genotypes (field entries) were grown in rows. The stems of the susceptible single-cross hybrid were highly covered with black teleutospores, whereas the TI selection remained completely disease-free

To further validate the markers and to demonstrate the applicability in breeding processes, plants with a homozygous resistance marker allele were intercrossed in isolation cabins (each population separately) and the harvested seeds were tested in the following year again in artificial inoculated field trials. There were no plants with disease symptoms for TI (Fig. 5) and HY75, and only a single susceptible plant in HY2407 (data not shown) could be found. Hence, the resistance was (almost) fixed in these populations.

## Discussion

### Experimental setup

One challenge of the LST was to score the most-relevant and identifiable categories with a proper scaling. We adapted the IT developed for stem rust resistance scoring in wheat (Stakman et al. 1962) because it combined the degree and type of infection into one scale. Previous studies have shown (Gousseau et al. 1985; Roelfs 1988) that the IT was influenced by environmental factors like temperature or light, so that our results are only valid for the reported conditions that we kept as constant as possible when testing the material. The infections were high enough to observe all different ITs. Including the same susceptible check variety in all analyses (hybrid cultivar ‘Palazzo’) allowed us to keep track of the infection level and as already been addressed in the methods section, we used the lowest IT of the susceptible check (2.5) as basis for classification into resistant and susceptible. The field experiments were scored as percentage of stem surface covered with urediniospores. Likewise to the LST, all results must be based on the environmental factors given in that location. Field inoculum was composed of five isolates, including the three isolates also used in the LST. Those additional isolates could have resulted in a difference between the field and the LST; however, they differed only in the pattern and not by new virulence on other differential lines compared with the combination of all isolates used in LST (Figure S1) and other breeding material did barely reveal any differences between those isolates (data not shown). For the resistance gene *Pgs1*, it was observed that the resistance in the field can reduce the infection almost to zero infection when no virulent isolate occurs (Gruner et al. 2020). However, full resistance of a plant in the field could also be the result of APR gene(s). In rye genetic resources consisting of cross-pollinating populations, we could expect all the different resistance types and combinations thereof (discussed later in more detail). As compromise of limited genotyping capacities but still aiming for mapping as much genes as possible in the genetic resources, we focused in this study on single R-genes that were detectable in the LST and have large effects in the field, i.e., classical all-stage resistances. This justifies our small population sizes of *n* = 73. With more populations tested (instead of larger populations) we could ignore the ones that had very rare alleles (WA) and those where probably several R-genes were segregating (HY75). In previous field tests (Miedaner et al. 2016), segregation into resistant and susceptible plants could be observed. In the field, we could not score ITs, because we used a mixed inoculum.

### Statistics

The cumulative logit mixed model was considered suitable for the LST data analyzed. The ordinal nature of the LST infection type could be used and codominant and dominant marker effects could be estimated by regression. Further, it allowed testing of several effects simultaneously (including a random term) and the results could be transformed in interpretable results. Given by the nature of isolate-specific resistances, it would had been ideal to extend our model by marker–isolate interactions, but the unbalanced distribution of ITs in combination with the marker scores and the small sample size in general resulted in convergence failure for many markers, so that additional isolate-specific models were fitted to not neglect this issue. The results from those models were compared with a results from a nonparametric test, and we showed that the same markers passed the significance threshold.

The threshold calculation proposed by Gao et al. (2008) was considered reasonable and easy to implement. Gao (2011) showed that the method produced almost equivalent results to a permutation test. However, a single step in the permutation procedure was based on the same test statistics as the final analysis. Thus, if the test statistics was biased in one direction, the final permutation threshold would shift in this direction and thus correct for the bias. The simpleM correction was only based on marker density and thus a correct test statistic was even more important.

The combination of isolates increased the number of data points and thus the power of the statistical testing. With single exceptions, all markers being significant across isolates were also detected for at least one isolate, but not every isolate-specific significance could be found across isolates. Because the sample sizes for a single isolate were one-third of the combined analysis and the measurements were not replicated on a single genotype, we considered the isolate-specific resistance signals with caution. This, however, impeded the clarification whether resistance detected across isolates was really active for all isolates separately or if there were isolates that had overcome the resistance already. Assuming that the seedling resistances could also be detected in the adult-plant stage, artificial inoculation with the same isolates in mixture as done in the validation experiment should have caused at least partially susceptible reactions on those plants. However, as we observed a high number of fully resistant plants, we concluded again that the missing isolate-specific resistances were a matter of small sample size and not of overcome resistances.

In addition, the estimation of the genetic variance was limited by the experimental design. Because only a single observation was made for each genotype-isolate (plant-isolate) combination in the LST, it was not possible to clearly separate between true genetic variance, isolate-specific reactions and experimental errors. Hence, also the estimated explained genetic variance was influenced by all different factors simultaneously.

### Analyzing self-incompatible populations

Our self-incompatible populations were based on improved landraces and pre-breeding material so that even several resistance genes could be segregating in a population. This, however, would even more hamper the detection of the single resistance genes. The difficulty compared to linkage mapping with additive and quantitatively inherited traits was that our IT scale was limited at both ends, and the allele frequencies of potential genes could range from zero to one. A single (dominant) gene that would be represented with high gene frequency in a population, could fully mask the resistance of another gene with low frequency, or spurious associations would be found that are caused by the joint distribution of two resistances. Models with marker combinations could (partially) address this problem, but it would involve more computational effort and the number of false positive associations would rise. The best would be to investigate populations with single (non-additive) genes or with quantitative traits and additively inherited genes, but especially in genetic resources every mode of inheritance must be expected.

The preselection step after the phenotypic analysis aimed to achieve higher significance and similar methods were also recommended for linkage mapping (p. 399f, Lynch and Walsh 1998). However, in cases with several segregating resistance genes this may be more important when the resistance gene is the rare allele. If we assume that several resistance genes are segregating in a population and the joint distribution would result in only a few susceptible plants, the selection toward susceptibility could also reduce the frequency of rare alleles masked by joint distribution, what would be counterproductive. As already addressed before, this is a major drawback of qualitative traits and we decided to adjust our mapping strategy on the assumption of single segregating genes and, consequently, also smaller population sizes but more populations to be tested. Further, selection in cross-pollinating populations leads to departures from HWE and thus restricts any marker filtering based on it. For non-quantitative inheritance and especially in the case of a dominant resistance gene, phenotypic selection from a larger population may require a larger number of phenotypically resistant genotypes to reach the same number of homozygous resistant alleles compared to unselected material in HWE (Figure S12). For this reason, we excluded the results from WA. The markers that could pass the significance threshold all segregated with three (two homozygous, one heterozygous) alleles, but this must (theoretically) result in a high number of resistant plants (Figure S12) and this was not observed in LST at least if resistance was defined as IT ≤ 2 (Fig. 1). In comparison, the selection in the other direction (phenotypically on the recessive allele), like done in HY2407, will directly increase the recessive allele.

### Marker linkage

Another factor that influenced the sample size required for mapping was the marker density and the linkage between markers (pp. 469ff Lynch and Walsh 1998). The marker density was considered high enough in our populations, because we found peaks with several significant markers (HY2407, TI) and the effective marker number that was required for calculating the critical threshold (p-value) was estimated to be only seven percent of the total number of polymorphic markers (Table S3). Further, the amount of linkage depends on the effective population size. Weir and Hill (1980) showed that linkage between two loci is inversely proportional to the effective population size. The maximum effective population size in our experiments was about 90 because of the previous use of isolation cabins for propagation with a maximum of 90 random mating plants. Further (unknown) multiplication or even breeding steps in the past could have reduced the effective population size even more and thus increased the linkage.

The disadvantage of the high linkage was that the significant markers were only significantly associated in the respective populations. For example, all markers listed in Table 3 were also segregating in all other populations, but without significant resistance association. This also shows that a mapping approach across all populations would be more challenging and would require much higher marker densities. Further, when we tried to validate the markers with single-plant testing in the field, some susceptible plants with resistant marker alleles were detected (Fig. 4). Under the assumption that the detected seedling resistance was also effective in the field, the linkage of marker and resistance was broken for those plants. The field-resistant plants with susceptible marker allele on the other hand could also be explained by incomplete linkage and additionally by adult-plant resistance that could, of course, not be tested in the LST.

### Field validation

When we tried to validate the KASP markers in field experiments, we were confronted with a highly unbalanced number of resistant and susceptible plants within the individual populations (Fig. 4b-f). The high frequency of resistant plants in the unselected material may have increased the probability of crossing resistance donors in isolation cabins so that the fixation of the three populations for resistance may have only partially been based on the marker selection and we must expect a high number of false positives. Ideally, we could define the IT in the seedling test that would correspond to the all-stage resistance observed in the field. With this information, the OSM1 measure, which gives the percentage of plants that fall into a certain IT category or below (we used IT ≤ 2), would be an appropriate predictor. Additional to the unbalanced number of resistant and susceptible plants, the presence of potential adult-plant resistance masked the count of false negatives. The separate analysis in seedling stage in the first place prevented us from observing more segregating resistances in a single population and thus reduced the risk of masking genes in the mapping analysis. From the accuracy point of view, we could show that also scoring of single adult plants could result in very high repeatability. This high repeatability was also confirmed by other field experiments located nearby (in the same year). However, instead of single plants, rows of inbred lines were scored (data not shown).

In our analysis, we included population HY75 that was used as resistance donor for *Pgs1* in a segregating inbred generation (Gruner et al. 2020). Unfortunately, we could not detect this gene in HY75 directly. However, we could fix the population for resistance with a *Pgs1*-linked marker from the other study. As discussed before, a potential presence of several resistance genes in a population and the small sample size may have impeded the identification of significant markers. Nevertheless, in HY2407 we could find a significant marker located at a similar position like *Pgs1*, so we used the combination of two markers for fixation of that population. The similar origin of both populations from the same breeding station in the Russian Federation could explain why we have detected the same locus.

### Resistances

In sum, we could identify three resistance loci. By using the markers from a previous study (Gruner et al. 2020), we referred one loci found in HY2407 to *Pgs1*. To the best of our knowledge, this has been the only stem rust resistance locus that has been characterized by markers and consequently we denominate the resistance gene found in population TI at the distal end of chromosome 1R as *Pgs2* and the one found in population OK at the distal end of chromosome 2R as *Pgs3*. There are several wheat varieties carrying translocations from rye with resistance against wheat stem rust. Noteworthy in regards to the location of *Pgs2* on the short arm of chromosome 1R and of *Pgs3* on the long arm of chromosome 2R are rye translocations in wheat with the same chromosomal segments and carrying stem rust resistance genes. Examples are *Sr31, Sr50* and *SrR*^*Amigo*^ located on 1RS and *Sr59* located on 2RL (McIntosh et al. 1995; Mago et al. 2015; Rahmatov et al. 2016). Without knowledge of a gene sequence, direct gene comparisons are difficult, but as those chromosomes have already been successfully used as resistance resources, the rye material studied here may also be an interesting resource for wheat breeding as the genes may also be active against *P. graminis* f. sp. *tritici*. Only few rye translocations are known in wheat that involved the chromosome 7R (Zeller and Koller 1981).

By using a LST in the first place, we were restricted to resistances expressed in the seedling stage. This type of resistance is mediated by single R-genes, mostly from the NBS-LRR (nucleotide binding site leucine rich repeat) class (Ellis et al. 2000). In rye, a reference sequence was published, but so far only a preprint publication is available (Rabanus-Wallace et al. 2019). Thus, we could not BLAST our sequences and check for linkage with potential candidate genes. But as shown by Rabanus-Wallace et al. (2019), several NBS-LRR-like pseudomolecules could be assigned to the distal ends of the chromosomes so that a BLAST would probably lead to several candidates. R-genes are often discussed in relation to concerns about durability because several genes of the R-gene class are known to be defeated by the pathogen (Ellis et al. 2014). This, however, needs also to be related to the crop. In contrast to self-pollinating crops, for rye it may also be possible to create cultivars (three- or four-way hybrids) with several segregating SR resistance genes and, in consequence, different sets of genes in the individual plant (Wilde et al. 2006). Practically, this could be handled either by using another gene for each component of three-way or four-way hybrid cultivars or by mixing the resistance alleles in population or synthetic cultivars. Theoretically, this should lead to a dilution and retarded emergence of virulent races, as the non-virulent race can still propagate on less-resistant plants of a cultivar. If a sexual cycle of the pathogen takes place this further dilutes the virulence of a pathogen on a genetic level. This idea is quite old and known under the term multilines in self-pollinating crops (p. 115ff in Miedaner 2011; p. 172ff Vanderplank 1984). However, it is not proven that this increase in resistance complexity of a cultivar will really be rewarded by long lasting effectiveness of resistances, and the effectiveness must be compared with pyramided resistances where every single plant of a cultivar has all resistance genes simultaneously (Vanderplank 1984). Additionally, the discussion on the durability of stem rust resistance in rye is highly speculative. To our knowledge, almost all German cultivars were susceptible to stem rust and only four newly released cultivars (KWS Gatano, KWS Eterno, KWS Binnto, KWS Edmondo) showed some or complete resistance. These were tested in a single location trial only (field test 2019, data not shown), so that almost no selection pressure was exercised on the pathogen until now. Given the low selection pressure in the recent past, it would also be interesting to directly release a cultivar with a high resistance gene complexity, or even complete pyramiding in all hybrid components, before the rust has already overcome the first single resistance gene. Practical examples of pyramiding or combining approaches of resistance genes in leaf rust showed that if lines with defeated resistances are combined, the disease level could be reduced but it did not lead to full resistance (Wilde et al. 2006). In case of hybrid breeding with three- and four-way hybrids, the inclusion of several resistance genes would demand a high breeding effort, but when using dominant genes the different resistance genes could be used in the different hybrid components.

### Application

As mentioned before, the markers linked with resistance in one population were also segregating in the other populations without the respective linkage so that marker-assisted breeding would also require a proper choice of crossing partners with the opposite marker allele to track the resistance. Marker-assisted resistance breeding may especially be interesting where two stem rust resistance genes are stacked in a breeding line and double stacks cannot be phenotypically separated from single genes in case both genes provide a full resistance.

The great advantage of the mapping approach used here is its simplicity and the low workload required. The random mating within the populations reduces unwanted population structure, like often observed in genome-wide association studies of self-pollinating crops. Even more important, no development of inbred lines is necessary that would take several years because a double-haploid approach does not routinely work in rye. We could show that it is possible to map single resistance genes directly in self-incompatible genetic resources. This gives valuable information before the introgression process has started. We presented statistical models suitable for ordered categorical data that are often generated in phenotyping seedling resistance. In particular, the isolate-specific modeling was mainly limited by the small population-wise sample size. If more complex traits are analyzed, the sample size must be increased, but the mapping must still be done within populations. Small effective population sizes positively influence the required marker density and thereby increase the chances to successfully detect highly linked markers. In sum, the proposed method can be used in a pre-breeding analysis prior to inbred line development in hybrid breeding or for direct selection in breeding populations or synthetic cultivars.

## Supplementary Information

Below is the link to the electronic supplementary material.

Supplementary file 1 (DOCX 3791 kb)
