# Supplementary material for: Mapping and validating stem rust resistance genes directly in self-incompatible genetic resources of winter rye
Source: Theor Appl Genet. 2021 Mar 10;134(7):1989–2003. doi: 10.1007/s00122-021-03800-7 (PMC8263455; doi:10.1007/s00122-021-03800-7)
Supplement: Supplementary file 1 — (DOCX 3791 kb) [file 122_2021_3800_MOESM1_ESM.docx]

# Mapping and validating stem rust resistance genes directly in self-incompatible genetic resources of winter rye - Supplement

Paul Gruner, Anne-Kristin Schmitt, Kerstin Flath, Hans-Peter Piepho, Thomas Miedaner

**Table S1** Virulence reaction of single pustule isolates (rows) on 15 rye differential lines (columns). The scores 0, 1 and 2 were given if no or only minor infections were present and the differential line (D) is regarded as resistant (no fill). If the score was 2.5, 3 or 4 differential lines were regarded as susceptible to reported isolate (grey fill). ^a^ Isolates were used additionally in a mixture with the other for field inoculation.

|  |  | **Differential lines** | | | | | | | | | | | | | | |
| --- | --- | --- | --- | --- | --- | --- | --- | --- | --- | --- | --- | --- | --- | --- | --- | --- |
|  |  | **D26** | **D31** | **D36** | **D30** | **D48** | **D25** | **D35** | **D47** | **D44** | **D37** | **D45** | **D41** | **D33** | **D46** | **D43** |
| **Isolates** | **3c-3** | 0 | 0 | 0 | 0 | 4 | 0 | 0 | 3 | 3 | 2 | 3 | 3 | 3 | 3 | 3 |
|  | **3h-3** | 0 | 2 | 0 | 3 | 0 | 3 | 2.5 | 2 | 2 | 3 | 2 | 4 | 4 | 3 | 3 |
|  | **43-1** | 0 | 0 | 0 | 0 | 1 | 3 | 4 | 2.5 | 2.5 | 4 | 3 | 4 | 4 | 3 | 2.5 |
|  | **3h-3**^a^ | 0 | 2 | 0 | 3 | 0 | 3 | 2.5 | 2 | 2 | 3 | 2 | 4 | 4 | 3 | 3 |
|  | **11-4**^a^ | 0 | 0 | 2 | 0 | 0 | 0 | 3 | 3 | 3 | 3 | 3 | 3 | 3 | 3 | 4 |

**Table S2** Flanking marker sequences and polymorphic SNP of the marker that were developed into KASP assays.

| **Marker** | **Left** | **SNP** | **Right** |
| --- | --- | --- | --- |
| isotig12934 | ACATACTTGTTTACAAGTATACTCCTCAGA | S | CTAKCTAGGCCGCAACACAAACCGTATCGC |
| C9750_251 | TACAYCATCAAAGAAAGGACAAAAAGGACA | M | GGCGAAGTTCTACAACTTTTTCTACGAAGC |
| isotig12866 | CGCTGTCGTTTCCGAAGACGGAAGGGACTT | K | CGTGGCAATCTTTGGCTTCTTGCTCATGTT |
| isotig14536 | CGTAAACTGTTGGCATTGTAGTACAATCTT | K | CGTGCAAGTAAAATAAGAAGAGCCAATCTT |

**Table S3** Overview of marker data. For all populations and the combination of all the number (N) of plants (genotypes) and markers, as well as the average heterozygosity and minor allele frequency (MAF) over all marker are reported. For each population markers were filtered to have a MAF > 5%, less than 5% missing values and at least three allele states. M_eff_ was calculated for p-value threshold adjustment in the mapping procedure.

| **Population** | **N plants** | **N marker** | **M_eff_** | **Heterozygosity** | **MAF** |
| --- | --- | --- | --- | --- | --- |
| All^a^ | 366 | 7641 | - | 32.6 | 28.9 |
| TI | 74 | 5958 | 400 | 37.0 | 29.1 |
| OK | 74 | 6045 | 427 | 40.2 | 30.6 |
| WA | 74 | 6040 | 448 | 39.7 | 30.3 |
| HY75 | 71 | 5907 | 387 | 38.0 | 29.0 |
| HY2407 | 73 | 6374 | 410 | 38.9 | 30.6 |

^a^ only filtered for missing values (<5%) and three allele states

**Table S4** Contingency table for population TI, marker isotig14536, and isolate Iso3c.3

|  | **0** | **1** | **2** | **2.5** | **3** | **4** |
| --- | --- | --- | --- | --- | --- | --- |
| **AA** | 3 | 8 | 0 | 2 | 0 | 0 |
| **AC** | 9 | 15 | 0 | 2 | 5 | 1 |
| **CC** | 0 | 0 | 2 | 11 | 13 | 3 |

**Table S5** Contingency table for population TI, marker isotig14536, and isolate Iso3h.3

|  | **0** | **1** | **2** | **2.5** | **3** | **4** |
| --- | --- | --- | --- | --- | --- | --- |
| **AA** | 5 | 6 | 0 | 1 | 1 | 0 |
| **AC** | 10 | 14 | 1 | 4 | 2 | 1 |
| **CC** | 1 | 0 | 6 | 8 | 12 | 2 |

**Table S6** Contingency table for population TI, marker isotig14536, and isolate Iso43.1

|  | **0** | **1** | **2** | **2.5** | **3** |
| --- | --- | --- | --- | --- | --- |
| **AA** | 3 | 8 | 0 | 1 | 1 |
| **AC** | 11 | 14 | 1 | 1 | 5 |
| **CC** | 1 | 1 | 2 | 14 | 11 |

**Table S7** Contingency table for population HY2407, marker isotig10644, and isolate Iso3c.3

|  | **0** | **1** | **2** | **2.5** | **3** |
| --- | --- | --- | --- | --- | --- |
| **AA** | 1 | 0 | 5 | 8 | 2 |
| **AG** | 16 | 5 | 2 | 2 | 3 |
| **GG** | 13 | 9 | 1 | 1 | 0 |

**Table S8** Contingency table for population HY2407, marker isotig10644, and isolate Iso3h.3

|  | **1** | **2** | **2.5** | **3** | **4** |
| --- | --- | --- | --- | --- | --- |
| **AA** | 0 | 7 | 7 | 2 | 0 |
| **AG** | 7 | 9 | 5 | 6 | 1 |
| **GG** | 10 | 7 | 3 | 4 | 0 |

**Table S9** Contingency table for population HY2407, marker isotig10644, and isolate Iso43.1

|  | **0** | **1** | **2** | **2.5** | **3** |
| --- | --- | --- | --- | --- | --- |
| **AA** | 0 | 1 | 6 | 6 | 3 |
| **AG** | 1 | 11 | 11 | 2 | 3 |
| **GG** | 0 | 17 | 5 | 1 | 1 |

**Table S10** Contingency table for population HY2407, marker isotig12866, and isolate Iso3c.3

|  | **0** | **1** | **2** | **2.5** | **3** |
| --- | --- | --- | --- | --- | --- |
| **AA** | 0 | 0 | 2 | 5 | 2 |
| **AC** | 14 | 9 | 2 | 3 | 3 |
| **CC** | 18 | 5 | 4 | 3 | 0 |

**Table S11** Contingency table for population HY2407, marker isotig12866, and isolate Iso3h.3

|  | **1** | **2** | **2.5** | **3** | **4** |
| --- | --- | --- | --- | --- | --- |
| **AA** | 0 | 3 | 6 | 0 | 0 |
| **AC** | 8 | 11 | 6 | 6 | 0 |
| **CC** | 10 | 10 | 3 | 6 | 1 |

**Table S12** Contingency table for population HY2407, marker isotig12866, and isolate Iso43.1

|  | **0** | **1** | **2** | **2.5** | **3** |
| --- | --- | --- | --- | --- | --- |
| **AA** | 0 | 0 | 3 | 3 | 3 |
| **AC** | 0 | 16 | 8 | 3 | 4 |
| **CC** | 1 | 14 | 11 | 3 | 1 |

**Table S13** Contingency table for population OK, marker C9750_251, and isolate Iso3c.3

|  | **1** | **2** | **2.5** | **3** |
| --- | --- | --- | --- | --- |
| **AA** | 0 | 11 | 13 | 5 |
| **AC** | 2 | 29 | 7 | 1 |
| **CC** | 0 | 4 | 1 | 0 |

**Table S14** Contingency table for population OK, marker C9750_251, and isolate Iso3h.3

|  | **1** | **2** | **2.5** | **3** | **4** |
| --- | --- | --- | --- | --- | --- |
| **AA** | 0 | 12 | 8 | 7 | 1 |
| **AC** | 3 | 20 | 7 | 3 | 0 |
| **CC** | 0 | 4 | 1 | 0 | 0 |

**Table S15** Contingency table for population OK, marker C9750_251, and isolate Iso43.1

|  | **1** | **2** | **2.5** | **3** |
| --- | --- | --- | --- | --- |
| **AA** | 2 | 5 | 9 | 13 |
| **AC** | 6 | 24 | 6 | 3 |
| **CC** | 1 | 1 | 2 | 1 |

**TableS16** Contingency table for population HY75, marker isotig12934, and isolate Iso3c.3

|  | **0** | **1** | **2.5** | **3** |
| --- | --- | --- | --- | --- |
| **CC** | 36 | 22 | 0 | 1 |
| **CG** | 4 | 1 | 1 | 2 |
| **GG** | 0 | 0 | 1 | 0 |

**Table S17** Contingency table for population HY75, marker isotig12934, and isolate Iso3h.3

|  | **1** | **2** | **2.5** | **3** | **4** |
| --- | --- | --- | --- | --- | --- |
| **CC** | 18 | 17 | 2 | 17 | 5 |
| **CG** | 0 | 2 | 0 | 3 | 3 |
| **GG** | 0 | 0 | 1 | 0 | 0 |

**Table S18** Contingency table for population HY75, marker isotig12934, and isolate Iso43.1

|  | **0** | **1** | **2** | **2.5** | **3** | **4** |
| --- | --- | --- | --- | --- | --- | --- |
| **CC** | 10 | 35 | 10 | 0 | 4 | 0 |
| **CG** | 0 | 4 | 0 | 0 | 2 | 2 |
| **GG** | 0 | 0 | 0 | 1 | 0 | 0 |

**Table S19** Contingency table for population WA, marker isotig18942, and isolate Iso3c.3

|  | **1** | **2** | **2.5** | **3** |
| --- | --- | --- | --- | --- |
| **AA** | 1 | 1 | 6 | 0 |
| **AT** | 0 | 11 | 24 | 1 |
| **TT** | 0 | 3 | 21 | 6 |


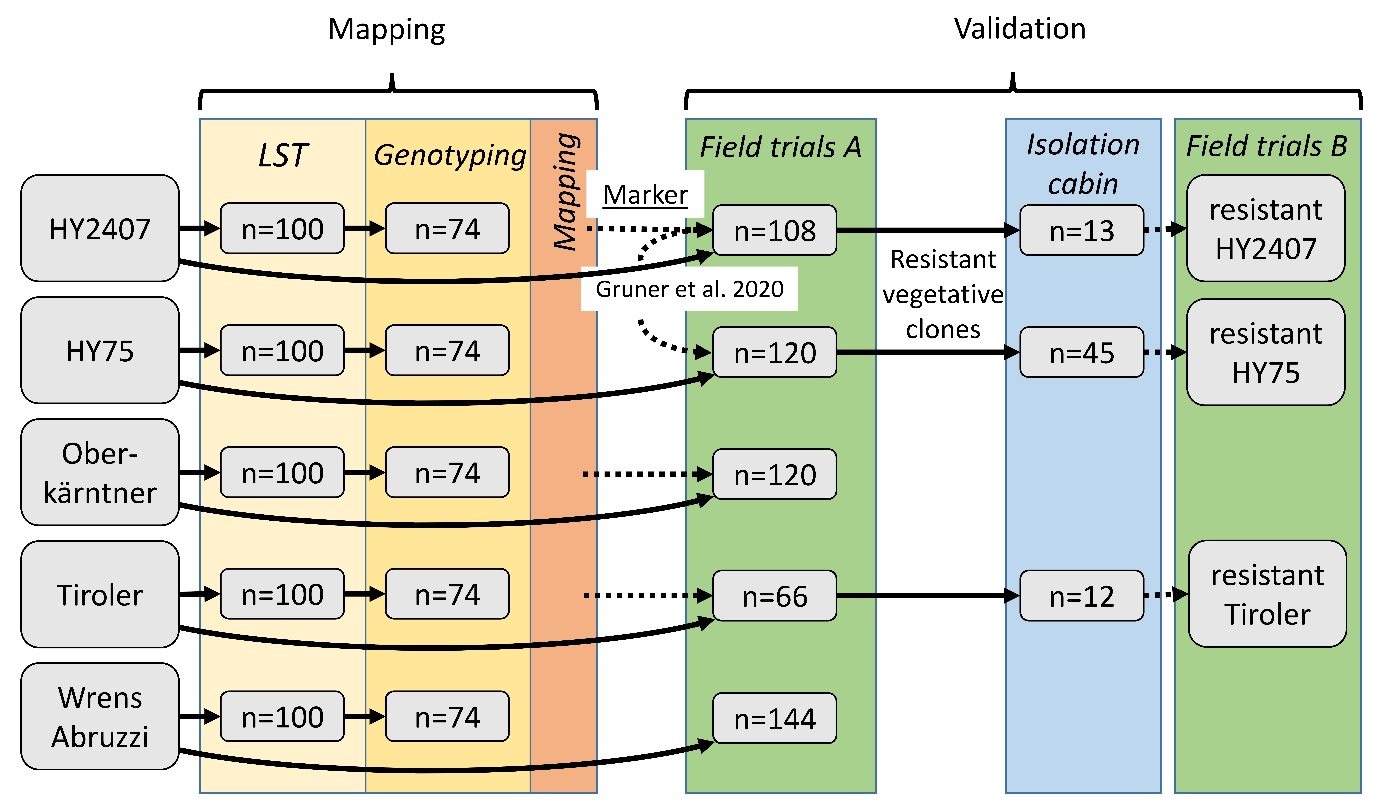


**Figure S1** Workflow of the study: Five self-incompatible rye populations (HY2407, HY75, Oberkärntner, Tiroler and Wrens Abruzzi) were investigated. In the first step 100 plants from each population were studied by leaf-segment test (LST) and based on the results 74 plants were chosen and genotyped with a 10K SNP chip. By combining phenotypic (LST) and genetic data the mapping of stem rust resistance was conducted aiming for significant marker-trait associations. Three promising markers (one each from HY2407, Oberkärntner and Tiroler) plus a marker from a previous study (Gruner et al. 2020) were chosen for conversion into a KASP assay and application on an independent seed sample of the same population. Those KASP-genotyped plants and non-genotyped plants from the remaining populations were investigated single plant-wise in an artificially inoculated field trial (Field Trials A). Before planting into the field, the plants were vegetatively cloned (ripped apart) to have replicates and to intercross the marker-based selected resistant plants in isolation cabins. The offspring of the intercrossed plants were again tested in field trials (Field trials B) in the following year. The marker chosen for ‘Oberkärntner’ could not successfully be converted into a KASP assay.


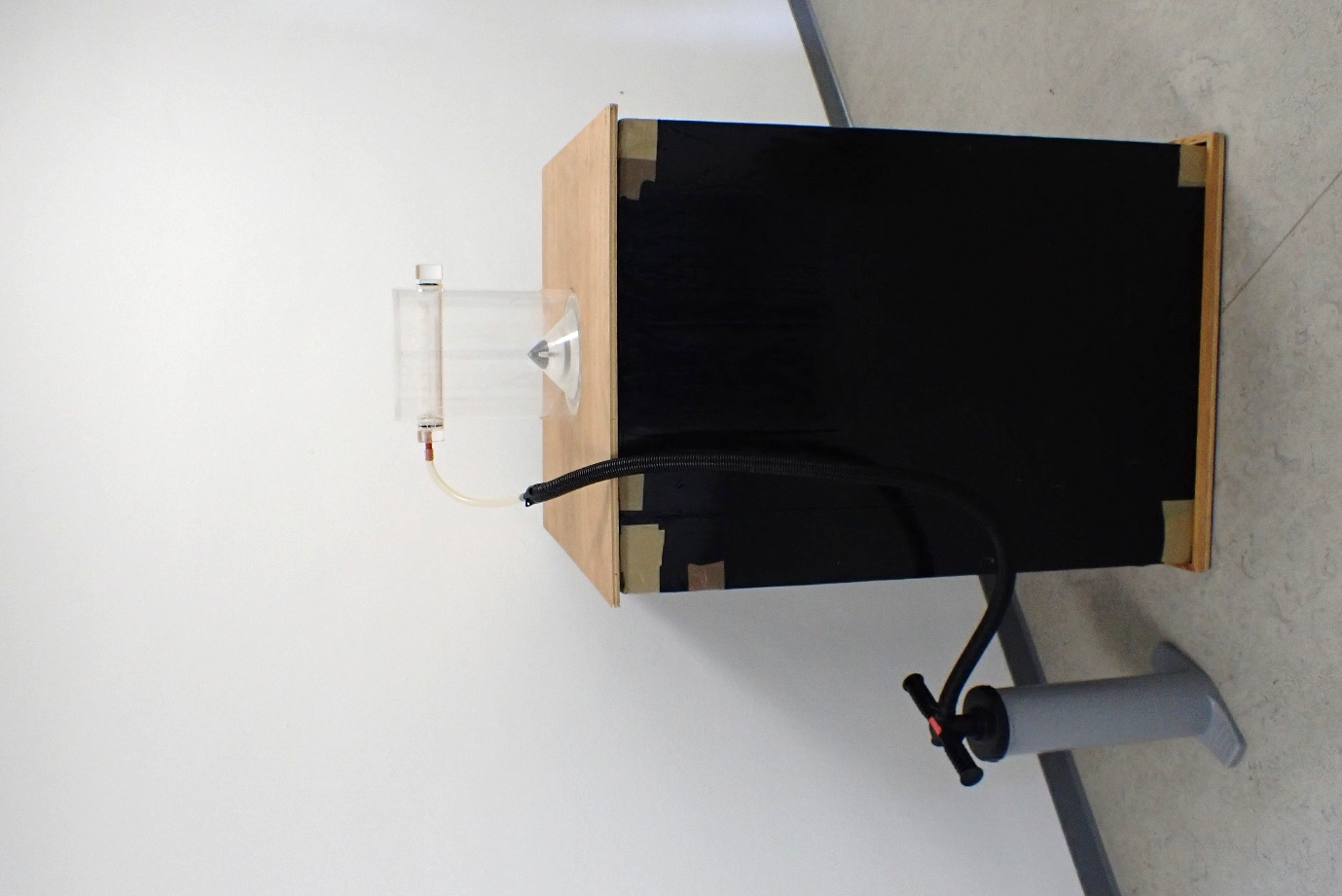


**Figure S2** The infection tower was used for inoculation of the multi-dishes. In this self-made device, the urediniospores were mixed with talcum powder in a ratio of 1:3 and filled in a perforated tube that was placed horizontally in top of an acrylic glass cylinder (top). The perforated tube was connected with a pipe of an air pump (left) so that the produced gust of wind blows up the spore-talcum powder and a fine dust was in the cylinder. The dust sank along an acrylic glass cone into holes in the top surface of a large box (center) were it further spreads out and sinks down onto the multi-dishes with leaf-segments on the ground of the box (not shown).


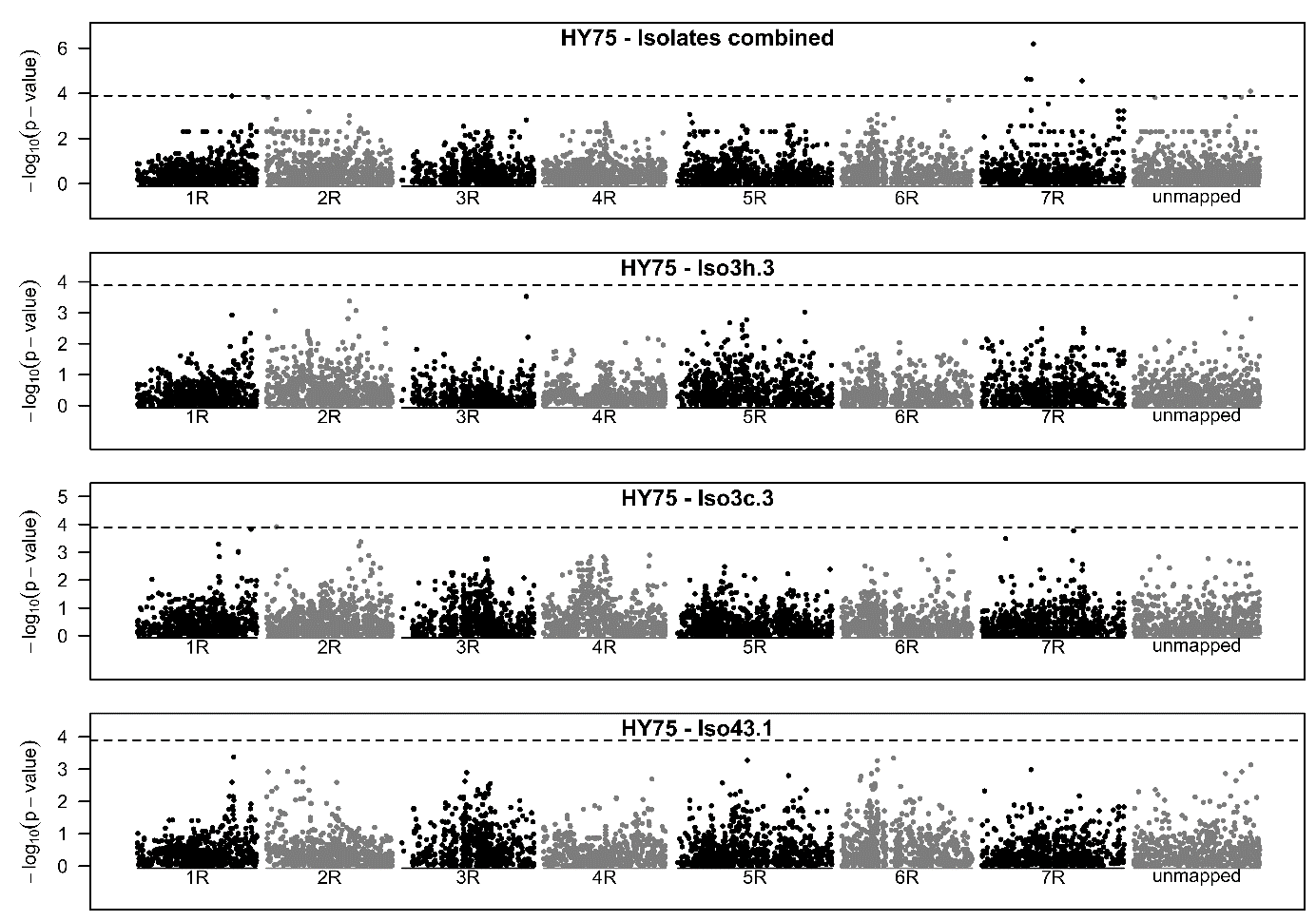


**Figure S3** Manhattan plot for marker-wise significance testing of association between infection type and SNP marker score of population HY75. A codominant (coded 0,1,2) and dominant (coded 0,1,0) marker effect was fitted simultaneously. P-values were based on ANOVA of the full model compared with a model without marker effects. The association was tested for all isolates combined (with fixed isolate effect and random genotype effect) and for all isolates (Iso3h.3, Iso3c.3, Iso43.1) separately.


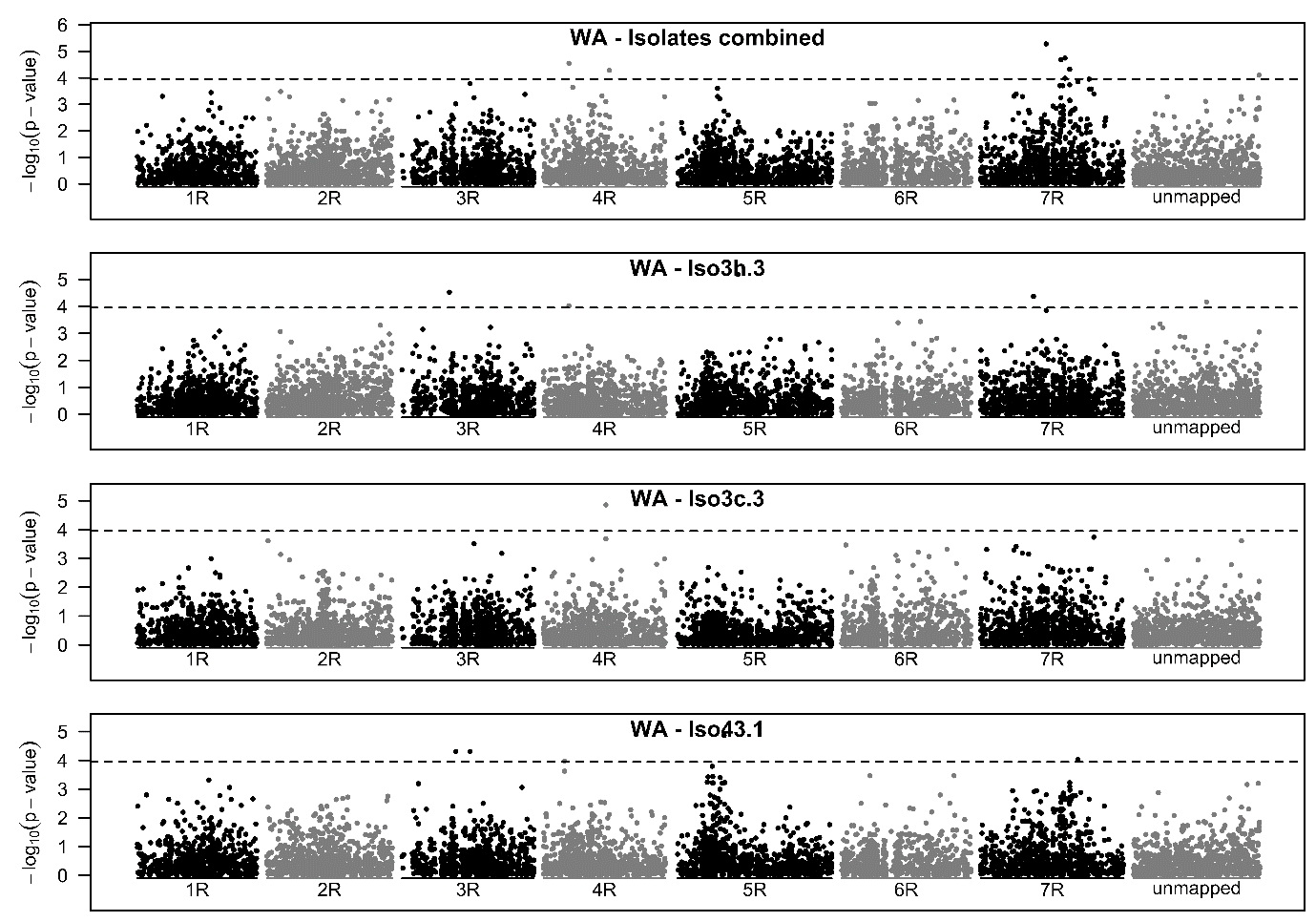


**Figure S4** Manhattan plot for marker-wise significance testing of association between infection type and SNP marker score of population Wrens Abruzzi (WA). A codominant (coded 0,1,2) and dominant (coded 0,1,0) marker effect was fitted simultaneously. P-values were based on ANOVA of the full model compared with a model without marker effects. The association was tested for all isolates combined (with fixed isolate effect and random genotype effect) and for all isolates (Iso3h.3, Iso3c.3, Iso43.1) separately.


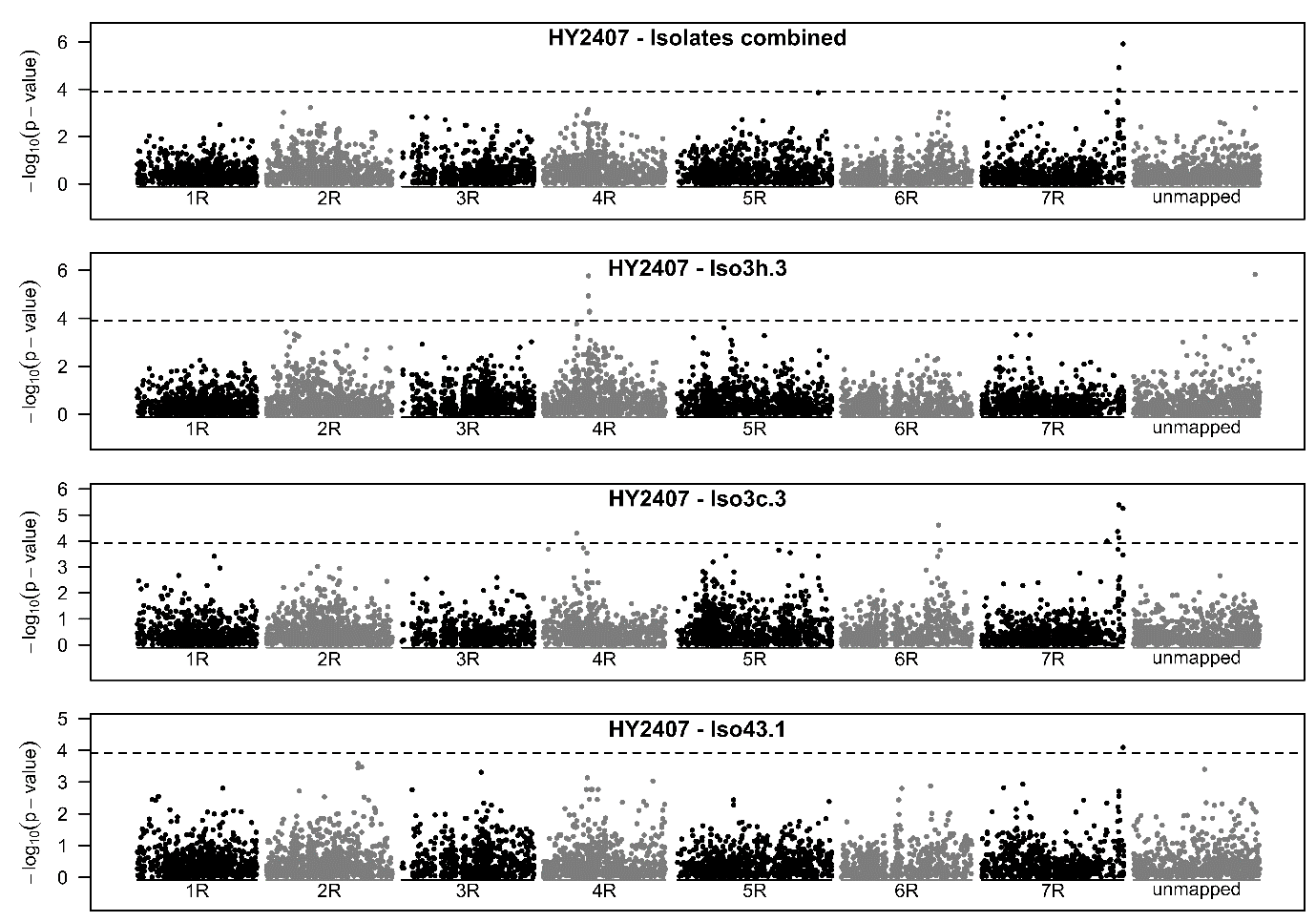


**Figure S5** Manhattan plot for marker-wise significance testing of association between infection type and SNP marker score of population HY2407. A codominant (coded 0,1,2) and dominant (coded 0,1,0) marker effect was fitted simultaneously. P-values were based on ANOVA of the full model compared with a model without marker effects. The association was tested for all isolates combined (with fixed isolate effect and random genotype effect) and for all isolates (Iso3h.3, Iso3c.3, Iso43.1) separately.


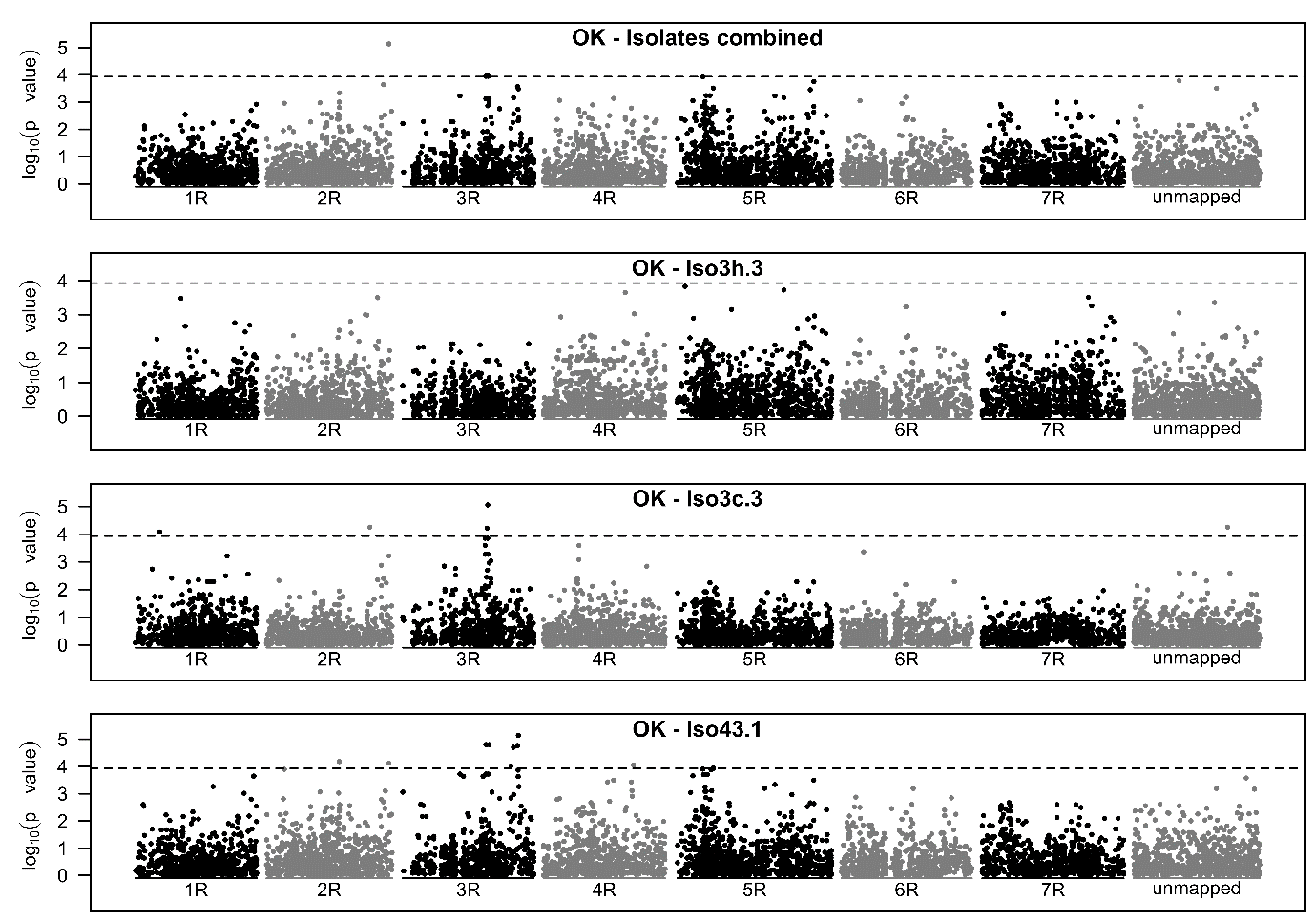


**Figure S6** Manhattan plot for marker-wise significance testing of association between infection type and SNP marker score of population Oberkärntner (OK). A codominant (coded 0,1,2) and dominant (coded 0,1,0) marker effect was fitted simultaneously. P-values were based on ANOVA of the full model compared with a model without marker effects. The association was tested for all isolates combined (with fixed isolate effect and random genotype effect) and for all isolates (Iso3h.3, Iso3c.3, Iso43.1) separately.


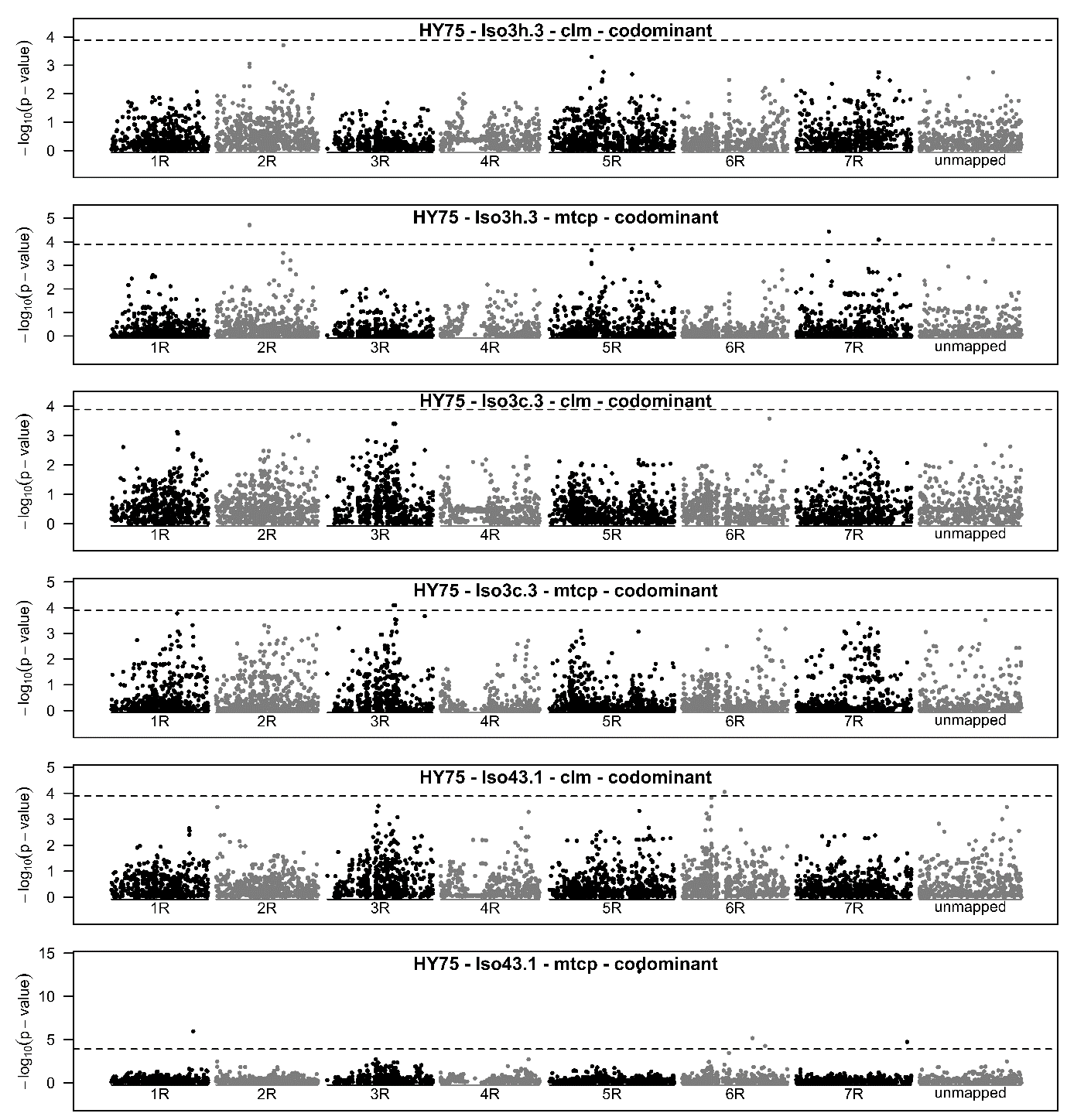


**Figure S7** Comparisons of clm model with non-parametric test fitted by mtcp() function for the population HY75 and the isolates Iso3c.3, Iso3h.3 and Iso43.1. For direct comparison only the p-values of the codominant effect (marker allele coded 0,1,2 in clmm and contrast -1, 0, 1 in mtcp) are shown.


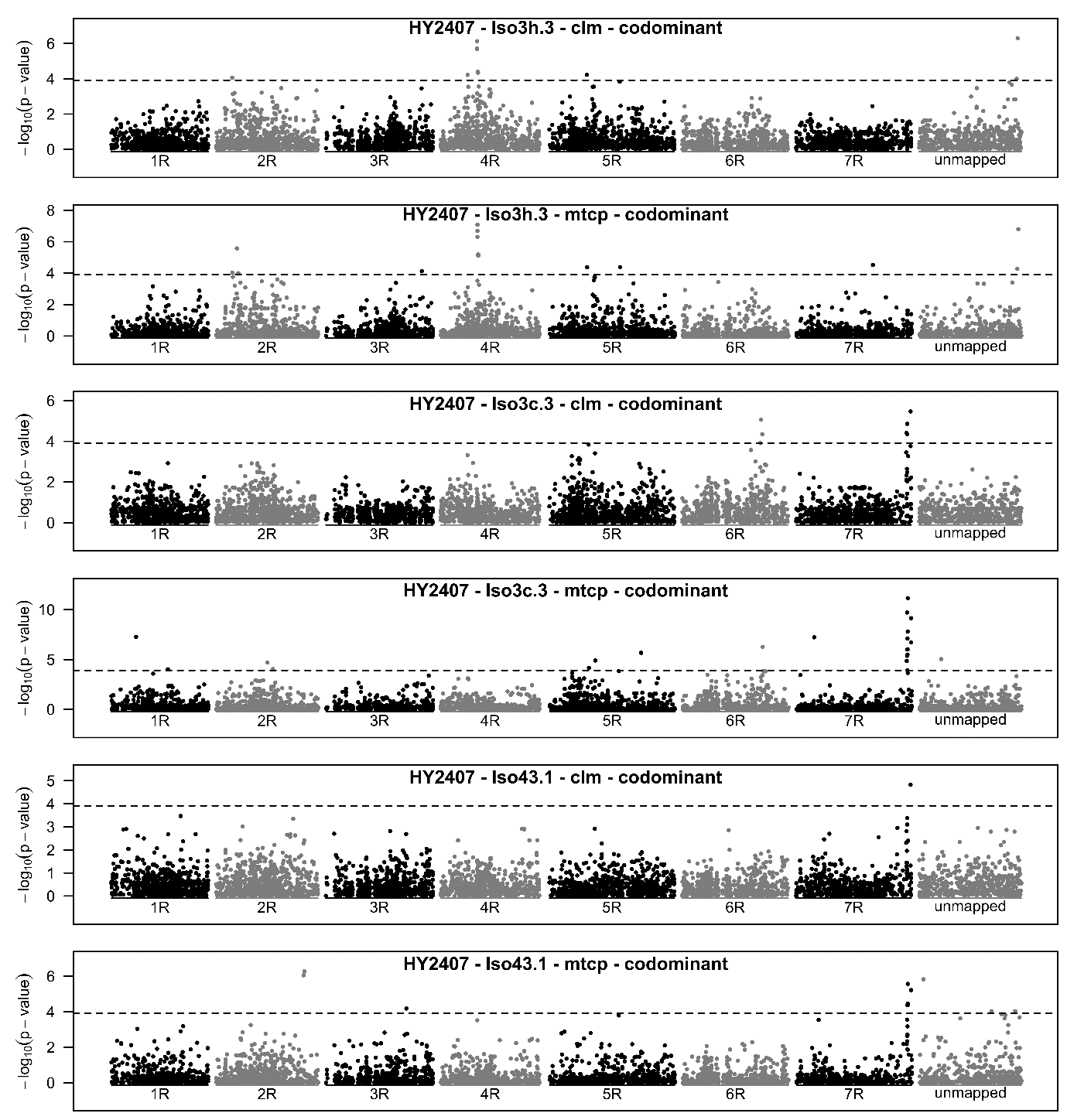


**Figure S8** Comparisons of clm model with non-parametric test fitted by mtcp() function for the population HY2407 and the isolates Iso3c.3, Iso3h.3 and Iso43.1. For direct comparison only the p-values of the codominant effect (marker allele coded 0,1,2 in clmm and contrast -1, 0, 1 in mtcp) are shown.


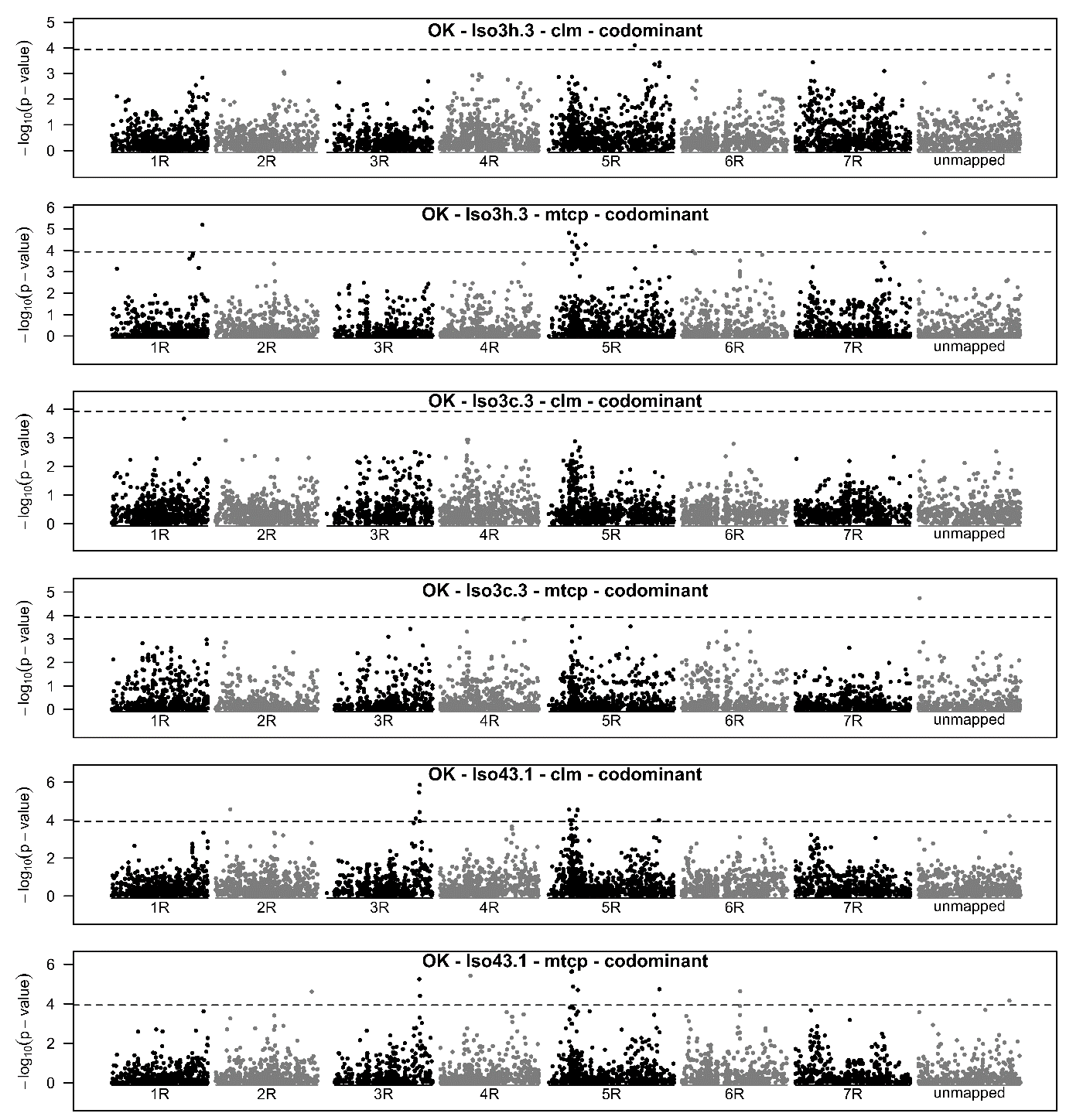


**Figure S9** Comparisons of clm model with non-parametric test fitted by mtcp() function for the population Oberkärntner (OK) and the isolates Iso3c.3, Iso3h.3 and Iso43.1. For direct comparison only the p-values of the codominant effect (marker allele coded 0,1,2 in clmm and contrast -1, 0, 1 in mtcp) are shown.


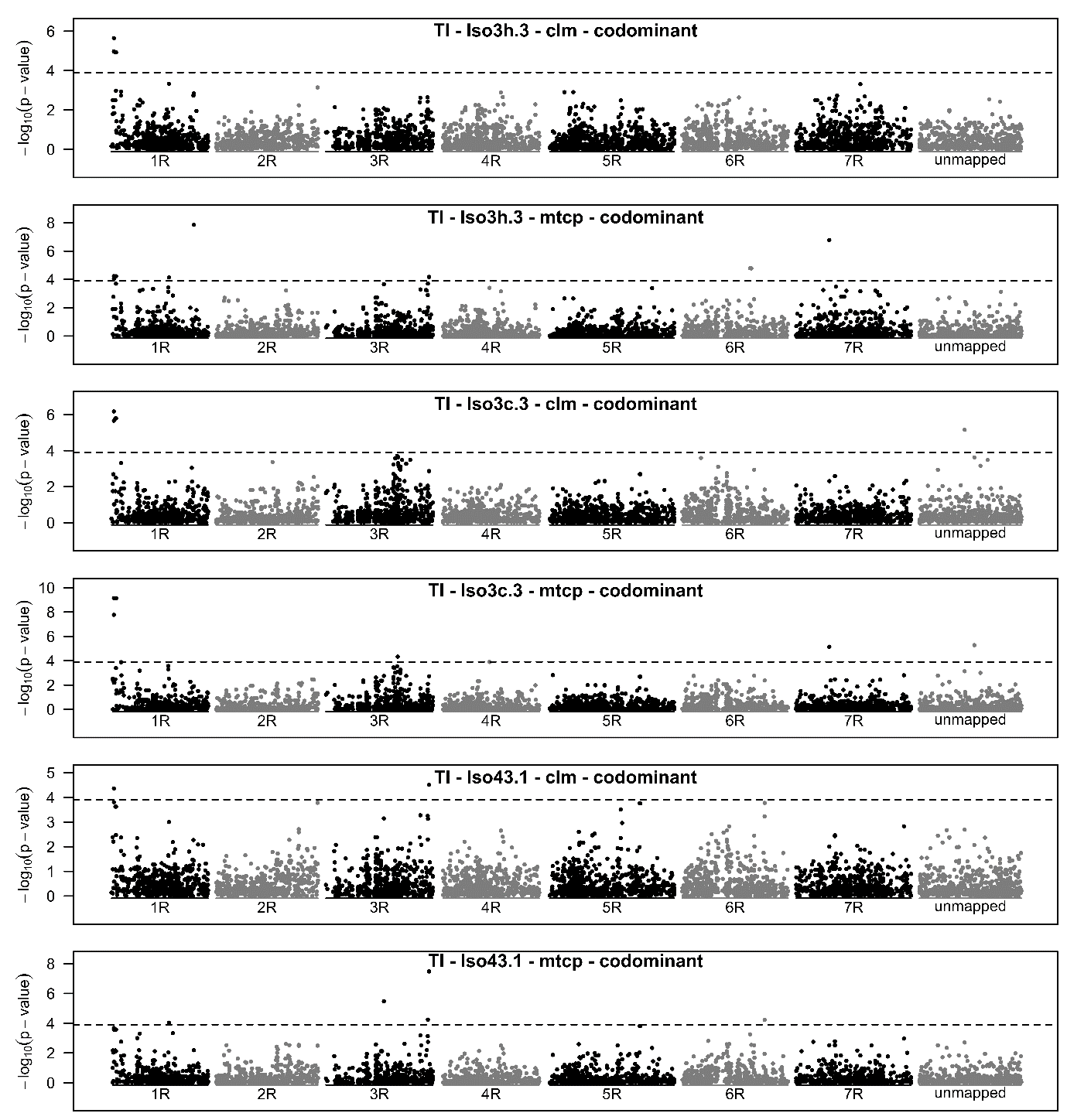


**Figure S10** Comparisons of clm model with non-parametric test fitted by mtcp() function for the population Tiroler (TI) and the isolates Iso3c.3, Iso3h.3 and Iso43.1. For direct comparison only the p-values of the codominant effect (marker allele coded 0,1,2 in clmm and contrast -1, 0, 1 in mtcp) are shown.


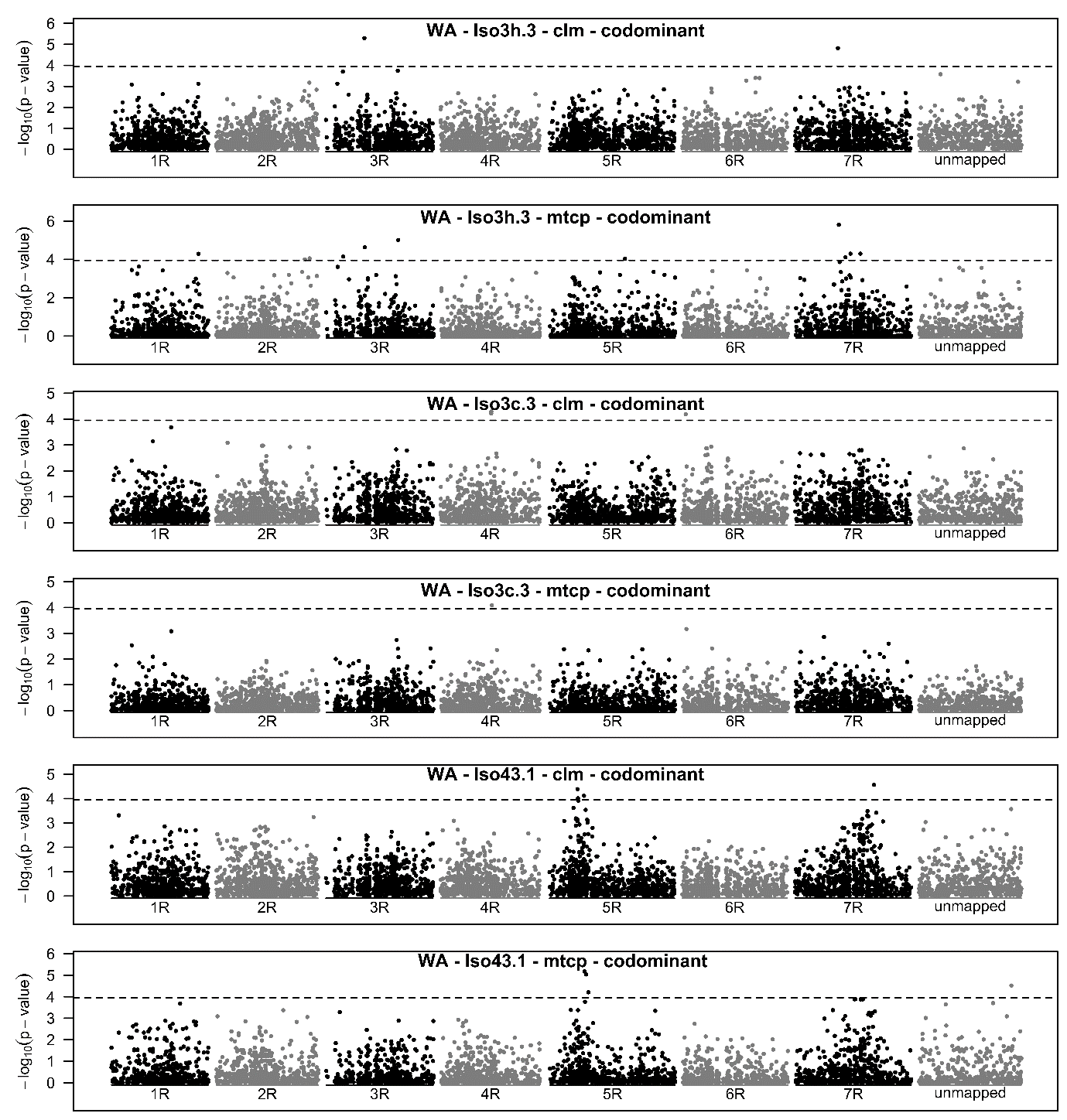


**Figure S11** Comparisons of clm model with non-parametric test fitted by mtcp() function for the population Wrens Abruzzi (WA) and the isolates Iso3c.3, Iso3h.3 and Iso43.1. For direct comparison only the p-values of the codominant effect (marker allele coded 0,1,2 in clmm and contrast -1, 0, 1 in mtcp) are shown.


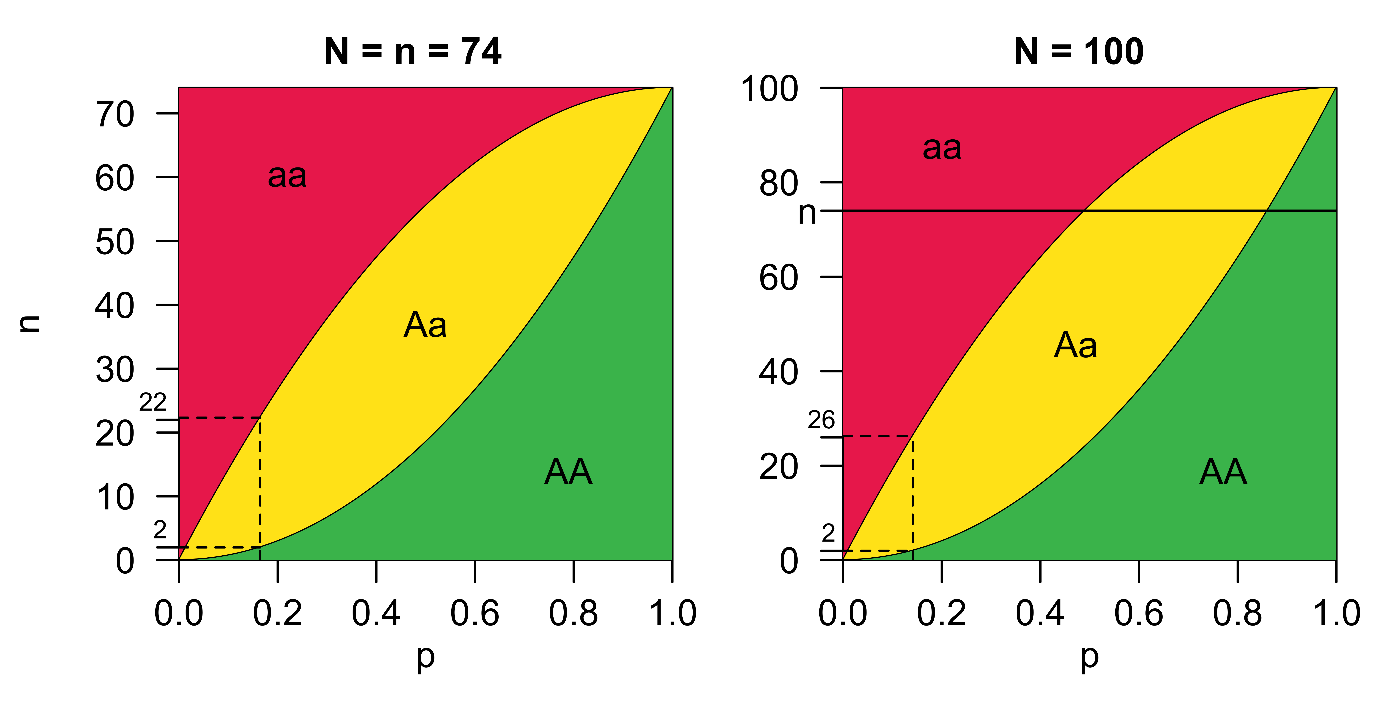


**Figure S12** Theoretical allele ratios for populations with N=74 (left) and N=100 (right) genotypes based on Hardy-Weinberg equilibrium (HWE). In the case that a resistance is dominantly inherited (allele A) the phenotypic ratios can become more balanced if a smaller population (n=74) is sampled from the larger population (N=100, right scenario), but this will influence the alleles ratios. Instead of having two homozygous aa alleles in 22 phenotypically resistant genotypes, 26 resistant plants would be required to also have two aa alleles.
